# Automation-aided construction and characterization of *Bacillus subtilis* PrsA strains for the secretion of amylases

**DOI:** 10.3389/fbioe.2024.1479626

**Published:** 2025-01-23

**Authors:** Felix Hamburger, Niels Schlichting, Michael Eichenlaub, Paul Igor Costea, Christopher Sauer, Stefan Jenewein, Johannes Kabisch

**Affiliations:** ^1^ Computer-aided Synthetic Biology, TU Darmstadt, Darmstadt, Germany; ^2^ BASF SE, Ludwigshafen, Germany; ^3^ Institute for Biotechnology and Food Science, Norwegian University of Science and Technology (NTNU), Trondheim, Norway

**Keywords:** automation, *B. subtilis*, amylase, secretion, chaperone, PrsA

## Abstract

Proteins face an obstacle race on their way to successful folding. Chaperones facilitate the proper folding of proteins by ensuring they remain on the correct path toward their final tertiary structure. In bacilli, the PrsA chaperone is essential for the correct folding and stabilization of proteins within the cell wall. Overexpression of the PrsA chaperone has been shown to improve the successful folding and secretion of many biotechnologically relevant secreted enzymes. This resulted in a double benefit: firstly, it promotes the efficient release of properly folded enzymes from the cell wall, and second, it reduces the folding stress for the cell, thereby enhancing the overall fitness of the production organism. This paper presents a workflow in which different wild-type PrsA molecules in *Bacillus subtilis* are co-expressed with different amylases having different signal peptides and promoters. To achieve this, six genome-reduced strains and nine PrsA proteins were systematically selected based on their cultivation performance and the production of two reference amylases. Following strain selection and deletion of major extracellular proteases, several hundred individual strains were created and screened using a stepwise and modular automation approach combined with amplicon sequencing. In addition to providing the key learnings from the workflow, it was revealed that no single PrsA molecule consistently improved amylase production, but genetic constructs combining different elements showed up to a 10-fold variation in yield. Among the screened constructs, the signal peptides YdjM and YvcE demonstrated the best performance.

## 1 Introduction

*Bacillus subtilis* is one of the most widely used organisms in biotechnology. It is the most studied Gram-positive organism with a genome sequenced in 1997 (Kunst et al., 1997; Earl et al., 2008). Its emergence as a model organism in biotechnology is supported by numerous processes that have received GRAS rating by the FDA and QPS assessment by EFSA (Leuschner et al., 2010; de Boer Sietske and Diderichsen, 1991). This includes its application in food production, such as historically in soy bean fermentation for thousands of years (Zweers et al., 2008). Because of their natural ability to secrete large amounts of homologous proteins like amylases and proteases into the medium and its good and prototrophic growth on cheap carbon sources, bacilli are generally used for the production of hydrolases (Gu et al., 2018). Secretion of the product is a major advantage in the industrial production of enzymes because it simplifies the purification process considerably in comparison with organisms that require lysis and separation from cytosolic host cell protein (Burdette et al., 2018) or possibly harbor undesired substances such as endotoxins in *Escherichia coli* (Petsch and Anspach, 2000). *B. subtilis* is readily genetically manipulable due to its ability to voluntarily absorb DNA through natural competence and integrate it into the genome. *B. subtilis* is now also used a heterologous expression system, although its ability to secrete non-homologous proteins has been described as being poor (Bolhuis et al., 1999). Yet some non-homologous amylase genes have been generated and secreted successfully since 1982 (Palva, 1982).

Amylases have a wide range of applications in the food, paper, detergent, biofuel, textile, pharma, and waste industries (Singh et al., 2016). Hence, they are among the most commercially interesting industrial enzyme classes. α -Amylases hydrolyze internal α 1,4-glycosidic bonds in polysaccharides, cleaving chains into short oligosaccharides (Elyasi Far et al., 2020). Nowadays, amylases are mostly produced by bacilli, and microbial amylases are generally superior over eukaryotic amylases as technical enzymes due to their inherent stability, which can be further altered and optimized for enzyme production or application using recombinant DNA technology (de Souza and e Magalhães, 2010).

*B. subtilis* produces a native α -amylase (AmyE), but only at low levels, and industrial processes may require a variety of amylases from other organisms, which are more thermostable, halophilic, or resistant to other harsh environments (Liu et al., 2009). Due to their industrial importance, several investigations have been conducted to improve expression, secretion, or folding using methods ranging from mutagenesis to fermentation optimization (Zhang et al., 2020). One way of enhancing amylase production is to increase the copy number of amylase genes in the genome (Son et al., 2016); however, if expression reaches a level where secretion and folding cannot keep up, enzymes might be degraded in the cytoplasm or cell wall (Wu et al., 1998). Hence, the host’s secretion and folding efficiency has to be increased when overexpressing amylase genes.

Secretion was shown to be a bottleneck in amylase production (Bolhuis et al., 1999; Yan and Wu, 2017). It can be generally split into three steps, namely, the cytosolic step, the membrane translocation, and the cell wall release and folding. In *B. subtilis*, proteins are generally secreted as pre-proteins with an amino-terminal signal peptide through the Sec pathway, aided by the signal recognition particle (SRP) and SecA (Kakeshtia et al., 2010), with the signal peptide likely acting as both a simple sorting signal for secretion and a folding factor, as described for *E. coli* (Park et al., 1988). Therefore, modifying the signal peptides is the most straightforward method that has been tried to optimize secretion (Freudl, 2018), but each step in the pathway harbors potential for improvement (Li et al., 2004). Although the cytosolic pathways (such as transcription, translation, and recognition by the secretion apparatus) are similar to those of other microbial hosts, the post-secretional processes in bacilli are unique, and the specific requirements have become obvious over the last few decades. It is now understood that post-secretional folding is important for successful cell wall release and that misfolding results in degradation at a cell wall-associated site, possibly before or during post-translocation (van Wely et al., 2001). In contrast, folding within the cytosol must be slow or even suppressed, with quick post-secretional folding being crucial to avoid aggregation and the stringent quality control found within the cell wall of bacilli. This kinetic partitioning has been initially described for *E. coli* with SecB as a holdase (Hardy and Randall, 1991). However, no functional SecB homolog has been described in bacilli. Therefore, alternative methods to enable kinetic partitioning are used. One example is the use of calcium ions to induce rapid folding: secreted enzymes fold efficiently with calcium addition into a protease-resistance form (Haddaoui et al., 1997), indicating that a low concentration of calcium in the cytosol might suppress folding, while a high level in the cell wall induce folding. Remodeling the cell wall has also been shown to improve post-secretional folding and impede with degradation (Vitikainen et al., 2005). PrsA, a cell wall-resident, membrane-bound chaperone, was found to increase amylase secretion in particular (Vitikainen et al., 2001; Vitikainen et al., 2004). It is the only chaperone in the cell wall of *B. subtilis* (Vitikainen et al., 2001). It has been described as the rate-limiting part of the secretion mechanism of α -amylases (Kontinen and Sarvas, 1993), and overexpression can enhance yield by supporting the secretion and folding of the secreted protein (Vitikainen et al., 2001). It appears to be important for amylases, although other exoenzymes such as protease benefit from elevated levels of this chaperone (Kontinen and Sarvas, 1993). Expressing heterologous amylases with the host’s native PrsA may not be the optimal solution as amylase secretion and folding might vary depending on the PrsA chosen. Specific amylase–PrsA combinations seem to be particularly synergistic (Quesada-Ganuza et al., 2019). Additionally, by finding an active amylase–PrsA pair, the *B. subtilis* secretion stress response can be decreased (Quesada-Ganuza et al., 2019), which, in turn, result in increased fitness and hence improved overproduction. It is worth noting that creating a specific pair of PrsA and suitable exoenzymes might not be straightforward in common cases such as 1) exoenzymes from metagenomes where the suitable chaperone is not known; 2) enzymes which are engineered and hence have an altered folding pathway over the parent; or 3) insufficient compatibility of the proteolytic quality control of the host with the heterologous PrsA molecules and degradation of the chaperone (Krishnappa et al., 2014).

Modern screening techniques might, therefore, be used to identify the optimal PrsA chaperone with the appropriate level in the cell wall to ensure that the secretion and folding processes are smooth and efficient. In this publication, we present the automation-aided construction of hundreds of *B. subtilis* strains with different combinations of the PrsA chaperones (varying the chaperone and the promoter used for its expression) and the expression cassette for amylases (varying signal peptides, promoters, and the enzyme). We describe the process from strain selection to the construction and analysis of different PrsA strains with model amylases, the construction of a multi-protease knock-out strain, and the automation-aided construction of amylase expression cassettes, as well as share the practical experience when using a robotic platform for cultivation and strain generation.

Using amylase activity as a readout, we found that some PrsA molecules are generally more efficient than others, hinting that neither the homologous PrsA of the host nor the homologous PrsA to the amylase will be the superior and right choice. We also discovered that modifying the signal peptide does not replace the action of PrsA in bacilli, implying that signal peptides might contribute to enhance folding but might not replace the foldase.

## 2 Results

### 2.1 Selection of expression strains

#### 2.1.1 Growth of parent strains

**FIGURE 1:**
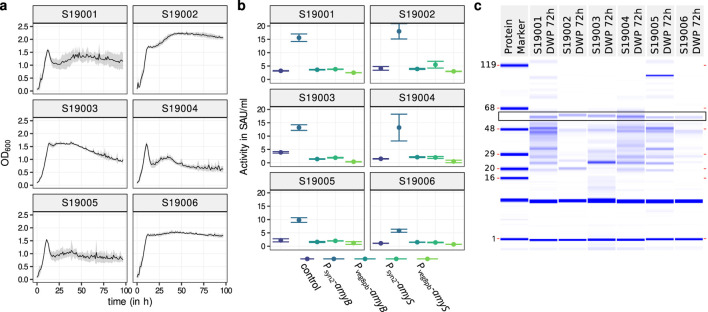
Selection of the initial strain. **(A)** Growth analysis of initial *B. subtilis* expression strains considered in this work (Supplementary Table S1). Cultivation was realized in 200- μ l flat bottom microtiter plates at 30°C and 1,000 rpm with seven replicates (S19006: n = 6). ${\text{OD}}_{600}$ was measured on line in 1-h intervals via a microplate reader. Standard deviation is shown as the gray background. **(B)** Amylase activity of strains with different amylase expression cassettes. ${\text{P}}_{\mathrm{syn2}}$ -*amyB*, ${\text{P}}_{\mathrm{veg8pb}}$ -*amyB*, ${\text{P}}_{\mathrm{syn2}}$ -*amyS*, and ${\text{P}}_{\mathrm{veg8pb}}$ -*amyS* with the control only containing the native *amyE* gene. Cultivation was done in deep-well plates at 30°C with 300 rpm (n = 8). **(C)** Exemplary LabChip measurements of expression of ${\text{P}}_{\mathrm{syn2}}$ -*amyB*-transformed strains. AmyB is highlighted with a black box.

A suitable expression strain should have robust molecular biology traits such as transformation efficiency, good growth with low lysis in expression media, and finally an efficient expression of the target molecule. Six different *B. subtilis* strain options (S19002 through S19007; see Supplementary Table S1) were contributed to the project. To select the best amylase production chassis, the growth profile of the respective strains was monitored over a cultivation of 96 h. The strains were cultivated in microtiter plates (MTPs) for continuous growth measurements using a robotic platform. Additionally, they were cultivated in deepwell plates (DWPs) to produce a model amylase. The DWP-cultivations have higher comparability when it comes to upscaling for industrial processes (Habicher et al., 2021). ${\text{OD}}_{600}$ was automatically monitored in MTP using a robotic platform described in the Methods section and depicted in Supplementary Figure S1, while with the DWP cultivation, the amylase activity was assayed, and lysis was estimated using a LabChip. The results are depicted in Figure 1. Until approximately 10 h after inoculation, the growth rates of the strains were very similar, with S19002 and S19006 reaching a slightly higher peak at optical density than the other strains. Directly after the transient phase, S19001, S19004, and S19005 cultures began to lyse. In contrast, S19003 experienced a steady decrease until the end of the 96-h experiment. S19006 and S19002 showed comparable lysis. S19002 showed another growth spurt to a measured ${\text{OD}}_{600}$ maximum of 2.25 at 50 h post-inoculation, where it entered the stationary phase. As the cultures were not diluted for ${\text{OD}}_{600}$ measurements, values above 2.0 in MTP are underestimated in our setup (see Supplementary Figure S2 for diluted end-point OD measurements of the six strains) because they are no longer within the linear range. Nevertheless, the curves provide valuable growth profiles, albeit this imprecision at higher values.

#### 2.1.2 Amylase activity

To determine the optimal strain for amylase expression, we integrated four different promoter–amylase combinations into the *amyE* locus of each strain’s genome. ${\text{P}}_{\mathrm{syn2}}$ was used as a synthetic strong, constitutive promoter and compared ${\text{to}}_{\mathrm{veg8pb}}$ , which is moderately strong. AmyB from *Bacillus licheniformis* (also known as AmyL or AmyS, UniProt ID P06278) and AmyS from *Geobacillus stearothermophilus* (UniProt ID P06279) were selected as reporter amylases. Both amylases were used frequently in the literature and are members of the microbial alpha-amylase family with 61% identity. The amylases were combined into the following four transformation cassettes via Golden Gate cloning and PCR amplification: ${\text{p}}_{\mathrm{syn2}}$ -*amyB*, veg8pb -*amyB*, ${\text{p}}_{\mathrm{syn2}}$ -*amyS*, and ${\text{p}}_{\mathrm{veg8pb}}$ -*amyS*. The transformed strains were then cultivated for 48, 72, and 96 h to obtain growth data and select a time frame for further experiments (Supplementary Figure S2). Since there were just marginal changes between 72 h and 96 h and to prevent possible lysis or excessive evaporation, the decision fell on a cultivation time of 72 h. A better growth of S19002 and S19006 could be observed, confirming the results shown in Figure 1A. For determining the suitability as expression strains, amylase activity assays were performed with DWP cultures harvested after 72 and 96 h. ${\text{p}}_{\mathrm{syn2}}$ -*amyB* had a considerably higher activity than all other promoter–amylase combinations (Figure 1B). This effect was observed in every tested strain, even though the amount of amylase activity differed in each strain. The maximum activity could be observed in S19002 (18 SAU/mL), while only one third of it could be observed in S19006 (5.8 SAU/mL). The other amylase cassettes were all at the same level as the controls, which had not been transformed and expressed as the native *amyE*. The base amylase activity of the strains also differed considerably from 1.1 (S19006) to 4.1 SAU/mL (S19002). Generally, *amyB* outperformed *amyS*, and ${\text{P}}_{\mathrm{syn2}}$ appears to be the superior promoter (1 b) and, thus, was chosen over the *veg8pb* promoter to drive amylase expression. To correlate the enzyme activity with amylase production and protein background, LabChip assays were performed on row A of the DWP cultures from 72 h to 96 h incubation time and the MTP from 72 h on the robotic platform. The abundance of the amylase was confirmed by the LabChip measurements, and additionally, it also shows that all strains except S19002 and S19006 are prone to lysis (Figure 1C).

All these results point to either S19002 or S19006 being the best choice of strain for further experiments because of good growth, high amylase activity, and weak protein background due to limited cell lysis. Although S19002 showed the overall best performance, it had the drawback that in contrast to S19006, the main extracellular proteases are not eliminated. In response to this strain, S19034 with deletions of these proteases was generated from S19002 in parallel to testing different promoter–prsA combinations in S19006, as described below. Full genotypes are listed in Supplementary Table S1.

### 2.2 PrsA tests

#### 2.2.1 Generation of *prsA* expression strains

First, the selected amylase cassettes ${\text{p}}_{\mathrm{syn2}}$ -*amyB* and ${\text{p}}_{\mathrm{syn2}}$ -*amyS* were transformed into S19006 and confirmed via colony PCR. Next, spectinomycin resistance was removed via Cre-Lox recombination using a transiently transformed plasmid carrying the *Cre* recombinase. Resulting strains were again confirmed via colony PCR and sequencing of the *amyE* integration site. For testing the effect, three promoters and nine *prsA* were selected to study the influence of different *prsA*–promoter combinations on amylase production in S19006. A list of *prsA* with their origins is given in Supplementary Table S2. The choice of PrsA molecules is based on the distance in the identity of the primary sequence, ranging from a distance of 85% in the case of *B. subtilis* and *Bacillus amyloliquefaciens* PrsA to approximately 40% identity in the case of *B. subtilis* and *Bacillus halmapalus* PrsA (see identity matrix in Supplementary Figure S14). Of the three promoters tested, the first two are constitutive ( ${\text{P}}_{\mathrm{secA}}$ and ${\text{P}}_{syn\text{\_}weak}$ ) and the last promoter (#5, ${\text{P}}_{\text{spac}}$ ) is activated by the addition of 0.1 mM IPTG. All promoters are mid- to strong promoters, according to the data provided at SubtiWiki (Pedreira et al., 2022); however, they are not predicted to create a strong burden on the cells. It is worth noting that ${\text{P}}_{\mathrm{secA}}$ is almost identical to the native ${\text{P}}_{Bsu_{p}rsA}$ . It was regarded critical that the promoters be either regulated by an inducer or constitutive in order to facilitate the transition from the MTP screening format, which may be limited by oxygen or metabolite restrictions to a larger scale. It was critical that the physiological state of the cell not be a factor in this transfer. PCR products of the Golden Gate-cloned constructs for transformation were generated and transformed into S19006-*amyB* and S19006-*amyS*. Eight colonies from the selective agar were transformed for each construct–strain combination, verified by colony PCR, and used for inoculating the pre-cultures of the cultivation experiments. These were used to make cryoplates for storage directly before inoculating the main cultures of cultivation experiments. All strains containing constructs with a specific promoter (3 through 5) were cultivated in one plate, after which the MTP or DWP was named. Strains containing promoter 5 were cultivated without DWP5/MTP5 and with IPTG (DWP5 IPTG/MTP5 IPTG). For an exemplary plate layout, see Supplementary Figure S3. ${\text{OD}}_{600}$ growth curves of all samples were recorded and are found in Supplementary Figures S7, S8.

#### 2.2.2 Cultivation of *amyB*-expressing strains

During the cultivation, growth curves were recorded for every well in the MTP. These resembled the growth of the parent strain shown in Figure 1A and were very uniform (Supplementary Figure S4 top). Although MTPs show roughly the same results as DWPs, generally, DWP results are more pronounced. In DWP, the highest ${\text{OD}}_{600}$ could be observed in strains carrying constructs with promoter 4 with ${\text{OD}}_{600}$ medians up to 4.9 with *prsA* 453, originating from *G. stearothermophilus*, and the worst growth was observed in induced promoter 5 strains, especially the promoter containing *prsA* 456, originating from *Bacillus lentus*, with a median at ${\text{OD}}_{600}$ 2.8. None of the PrsA appeared to influence growth.

#### 2.2.3 Cultivation of *amyS*-expressing strains

As with *amyB* strains, growth curves recorded in *amyS* strains also closely resembled those of the parent strain S19006 (Figure 1A), with relatively minimal standard deviation (Supplementary Figure S4). Strains cultivated in DWP generally reached a higher ${\text{OD}}_{600}$ than those in MTP, but differences in growth, especially between promoters, are still observable in MTP. The total growth of *amyS* strains is comparable to that of *amyB* strains (Supplementary Figure S4), but the growth levels between promoters differ slightly. The ${\text{OD}}_{600}$ levels are much closer to one level, and promoters 3 and 4 are almost the same, with promoter 4 slightly ahead of promoter 3. Promoter 5 exhibits less growth, with IPTG-containing cultures growing slightly worse than uninduced cultures.

#### 2.2.4 α -amylase activity of *amyB* strains

For measuring the amylase activity, MTP and DWP culture plates from the cultivation experiments were frozen after the end of the experiment and tested for α -amylase activity, as described in Methods. Measurement results of these tests are shown in Supplementary Figure S5, upper panel. In contrast to the cultivations of the *amyS* strains, particularly for the MTP-based cultivations and to a lesser extent for the DWP cultivation, there is a significant difference between the eight replicates to the *amyB* strains compared to the *amyS* strain cultivations. In terms of the change in expression in the DWP measurements compared to the control, promoters 3 and 5 perform best, while promoter 4, which is encoded in the best growing strain, appears to be lagging behind. Heterologous *prsA* expression increased the *amyB* activity level compared to the control consistently. Particularly, high activities could be observed in the strains producing PrsA 449 (*Bacillus megaterium*) and 453 (*G. stearothermophilus*). The lowest amylase activity was measured in the strains transformed with *prsA* 457 (*B. halmapalus*).

#### 2.2.5 α -amylase activity of *amyS* strains

The activity data on *amyS* strains, on the other hand, are much more consistent (Supplementary Figure S5), but *amyS*, as previously observed (Figure 1B), shows a much lower amylase activity. DWP and MTP cultures have a nearly identical profile for the same PrsA in terms of final optical density. Contrary to the *amyB* strains, promoter 5 appears to be the one with the lowest activity results compared to promoters 3 and 4. When looking at the distance between control and PrsA strains, all PrsA except 457 provide an increase in *amyS* activity. PrsA 448, 452, and 453 have the highest activities, which are also among the top *amyB* strains. Peak activity measured in *amyS* samples with promoter 3 and PrsA 453 only amounts to 3.5 SAU/mL, while *amyB* activity values went up to 7.6 SAU/mL in DWP5 PrsA 453. In these experiments, promoter 3 provided the highest amylase activity increase compared to the controls in *amyB* and *amyS* strains, particularly in combination with PrsA 453, as well as PrsA 448, 449, 452, and 454. The PrsA conferring the lowest activity in combination with each promoter was 457, and its expression sometimes results in lower activity than in the controls (*amyS* strains and *amyB* MTP).

#### 2.2.6 Protease knockouts in S19002

It was determined that S19002 was the strain of choice for the final experiment of combinations of amylase, PrsAs, signal peptides, and different promoters because its growth test, amylase expression, and transformation efficiency test results were better than those of S19006. As the lack of protease knockouts was the main argument against using S19002, the strain was modified accordingly. Deletion plasmids for the sequential knockouts of *aprE*, *nprE*, *mpr*, *nprB*, *vpr*, *bpr*, and *epr* were constructed. Homology regions were used for the replacement of the respective protease gene with a spectinomycin resistance gene flanked by lox-sites (lox-SSS cassette) via homologous recombination. Following successful integration, the lox-SSS cassette could be activated by inducing the genomically integrated *Cre*-recombinase, which removes the *specR* gene via site-specific recombination, leaving a lox72 scar, where the protease coding region was before. Each knockout was confirmed by sequencing the respective site. The resulting final strain was named S19034 and verified via whole-genome sequencing, confirming that no genomic rearrangement as a result of the 20 consecutive Cre-Lox reactions and lox72 scars occurred in the generation of this strain. The sequencing revealed 33 point mutations leading to frame-shifts described in Supplementary Table S5. Several of these are annotated with functions relating to sporulation, including the sporulation essential sigma factor E, as well as proteins involved in cell wall functions. S19034 was then transformed with different promoter–*prsA* PCR cassettes described before. Transformation success was determined via colony PCR and sequencing. Successfully transformed strains were stored as cryostock. The generated strains with the origin of PrsA and strain names are summarized in Table 1.

**TABLE 1:** Promoter–*prsA* combinations generated in strain 19034.

Promoter	prsA gene	Organism of origin	Strain #
Promoter 3	*prsA* 447	*Bacillus licheniformis*	S19038
Promoter 4	*prsA* 447	*Bacillus licheniformis*	S19039
Promoter 3	*prsA* 448	*Bacillus amyloliquefaciens*	S19040
Promoter 4	*prsA* 448	*Bacillus amyloliquefaciens*	S19041
Promoter 3	*prsA* 452	*Bacillus pumilus*	S19042
Promoter 3	*prsA* 453	*Geobacillus stearothermophilus*	S19043
Promoter 4	*prsA* 453	*Geobacillus stearothermophilus*	S19044
Promoter 3	*prsA* 456	*Bacillus lentus*	S19045
Promoter 4	*prsA* 456	*Bacillus lentus*	S19046
Promoter 3	*prsA* 457	*Bacillus halmapalus*	S19047

### 2.3 Preparation of automatic cloning

#### 2.3.1 Construct selection

In total, 28 different amylases have been chosen from the “Termamyl-like” amylases of the GH13 family as the target sequences (Drula et al., 2022). Members of this family are used commercially in the food, biofuel, or detergent industries and are named after the first commercial product from this family (Termamyl L; introduced by Novo Nordisk in 1973). Members of this class are within a sequence range of 60% and display a core three-domain structure composed out of an interwoven A/B domain with an adjacent C-domain. Common is an unusual Ca^2+^–Na^+^–Ca^2+^ triad at the A/B-domain interface next to the substrate-binding site (Machius et al., 1995).

It was planned to transform a set of nine *prsA* with either promoter 3 or promoter 4 into the base strain S19034. These strains should then be transformed with the 28 different amylases, coupled with two promoters and four signal proteins. In total, 2,240 different combinations of PrsA, amylase, amylase promoter, and signal peptide were tested in a single run. As automation-supported strain generation of this scope needs careful preparation, we took into account the following aspects before starting with actual strain generation.

#### 2.3.2 Positional effects in the MTP incubator

During the previous experiments, observations showed that cultivations in MTP, which were performed in the Cytomat2 tower shakers, yielded less precise results than the cultivations in DWP, which were incubated in custom-designed humidity boxes (Bruder et al., 2019) in regular incubator shakers. Evaporation, particularly in wells at the edge of the MTP, appeared to be a cause of variation. This observation led to the question how big the positional effects already observed in other experiments actually are. To investigate this problem, a set of MTPs was grown together with the *amyS* strain with different *prsA* genes in various positions on the incubator shaking towers. A measure of 200 mL medium was inoculated with 2 mL of S19006 pre-culture and distributed into six MTPs with 200 μ l per well. ${\text{OD}}_{600}$ was measured, and MTPs were placed in the shaking tower and incubated at 30°C at 1000 rpm. After 72 h, the MTP were taken out of the incubator, and ${\text{OD}}_{600}$ was measured at a 1:20 dilution. After the experiment, a decrease in the volume was noticed in the wells with higher ${\text{OD}}_{600}$ , ranging from mostly slight to significant (Position 13). Supplementary Figure S6 shows the ${\text{OD}}_{600}$ increase of every well in every MTP from the start to the endpoint measurement.

It was observed in all positions that the higher column number H row always shows higher ${\text{OD}}_{600}$ than the rest of the wells. This pattern can also be observed in outliers of the previous experiments (Supplementary Figure S4, top graph, MTP row). The ${\text{OD}}_{600}$ of the MTP in position P14 additionally has a lot of singular outliers from rows A to C. General measured ${\text{OD}}_{600}$ of all wells appeared to increase when moving further to the top positions in the towers (Supplementary Figure S6). This is particularly evident at position 13, where the G row behaves similarly to the H row in all other MTP, resulting in a significant volume decrease. As a consequence for this work, column H was filled with water instead of samples, and the lower position P14 was not used anymore.

#### 2.3.3 Transformation tests

**FIGURE 2:**
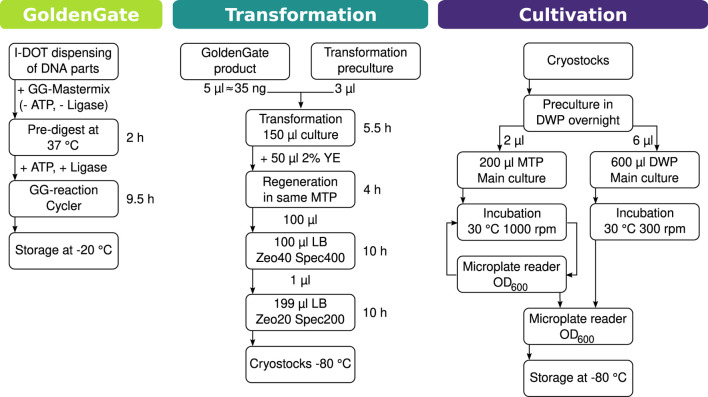
Chart of the workflow for automated cloning, transformation, and cultivation. The workflow is divided into three parts, after any of which pausing the process is possible. I.DOT is a contactless nanoliter dispenser. A more extensive version of this workflow can be found in Supplementary Figure S9.

Since the *B. subtilis* transformation protocol described in the methods cannot be readily automated in MTPs, a new method of transforming it exclusively in liquid media had to be developed. A particular problem of downscaling the transformation in a fully automated manner is the plating onto antibiotic-containing agar plates for a selection of transformants. This could not be readily accomplished by the robotic platform, especially not in the required scale. The solution found is to carry out the selection process by continuous cultivation in liquid selective media, as shown in the scheme in Figure 2. In order to determine all the parameters necessary to reduce the transformation to MTP size and make it automation-compatible, manual tests were carried out in MTP. First, it had to be determined whether the transformation will work in such small volumes with different shaking parameters and therefore changing oxygen supply. At the same time, we wanted to test whether amplifying the Golden Gate product with PCR would yield better results. To determine this, an experiment was set up with the genetic material from the initial amylases test ( ${\text{p}}_{\mathrm{syn2}}$ -*amyB*, see Section 2.1.2). Results are presented in Table 2, which shows the number of wells with growth in the final culture MTP. Successful transformation was then confirmed via colony PCR. None of the negative controls grew, and only one well that should have been transformed remained unchanged. This was the well where spectinomycin was added directly during the regeneration phase.

**TABLE 2:** Tests for determining transformation mix and liquid selection variables. Growth in MTP wells. −ctr describes transformation mixes without DNA, and + ctr represents positive controls transformed with 85 ng p19009.

Golden Gate product vs. PCR cassette						
Yeast extract regeneration				Yeast extract Spec200 regeneration		
Transformed	− ctr	PCR	GG	+ ctr	PCR	GG
	0/3	3/3	3/3	3/3	3/3	2/3
− ctr	0/3	0/3	0/3	0/3	0/3	0/3
Antibiotic concentration						
Transformed	Spec100	Spec200	Spec300	Zeo20	Zeo20	Zeo20
				Spec100	Spec200	Spec300
	2/3	3/3	3/3	3/3	3/3	3/3
− ctr	1/3	0/3	0/3	0/3	0/3	0/3
Antibiotic controls						
Transformed	LB5	Zeo20	Zeo10 Spec100	+ ctr Zeo20 Spec300		
	3/3	3/3	3/3	3/3		
− ctr	3/3	3/3	1/3	0/3		
Regeneration test						
Transformed	3 h YE	2 h YE	no YE	3 h YE	2 h YE	no YE
				Spec100	Spec100	
	3/3	3/3	0/3	3/3	3/3	0/3
− ctr	0/3	0/3	0/3	0/3	0/3	1/3
Golden Gate product concentration						
Transformed	0 μ l	0.75 μ l	1 μ l	1.5 μ l	2 μ l	3 μ l
	0 ng	24 ng	32 ng	48 ng	64 ng	96 ng
	0/3	3/3	3/3	3/3	2/3	1/3
− ctr	0/3	0/3	0/3	0/3	0/3	0/3

The following transformation tests were conducted with a few adaptations. To facilitate pipetting on the robotic platform, the addition of 50 μ l of 2% yeast extract was tested (instead of the more concentrated 10% stock). Since it is critical to replicate experimental conditions as closely as possible, they were incubated in the robotic platform at 37°C at 1000 rpm and only taken out for automatic ${\text{OD}}_{600}$ measurements every hour and liquid handling, which was done manually. S19038, one of the strains with an integrated *prsA*, was used for transformation. The test of different antibiotic concentrations showed some growth in Spec100 and Spec100+Zeo10 controls. Hence, a higher concentration, LB5 Spec200+Zeo20, was chosen for the workflow to enable efficient selection and prevention of contamination. Different regeneration times were tested as well, with yeast extract being added 2 or 3 hours after inoculation of the following culture, with or without spectinomycin. Having no regeneration time was lethal for transformants, and no difference was observed between the yeast extract with and without spectinomycin. The final experiment in this series involved the addition of a Golden Gate reaction mix. The concentration of successfully assembled plasmids was determined by agarose gel analysis, and different concentrations of DNA were tested for the transformation of S19038. It appears that higher DNA concentrations reduce the transformation efficiency as only 2/3 of transformations with 64 ng and only 1/3 of transformations with 96 ng Golden Gate products were successful. Thus, 1 μ l (approximately 32 ng) was chosen for the workflow because smaller volumes are difficult to pipette and may result in some pipetting inaccuracy.

#### 2.3.4 Workflow for automated cloning and cultivation

A workflow was designed to set up the automated cloning of strains on the robotic platform (see Figure 2). The procedure was split into three parts: creation of gene construction, transformation, and cultivation. Following each of these, the workflow can be suspended. For the cloning of the necessary gene constructs, it was decided to use the Golden Gate method because of its modularity and flexibility. To use as little DNA parts as possible, a nanoliter dispenser (I.DOT, Dispendix) was used for dispensing the Golden Gate parts into a 96-well PCR plate. Based on the data gathered in transformation experiments (Table 2), the workflow for the automatic transformation of *prsA-*transformed *B. subtilis* strains was designed. Transformation parameters were set to 1.5 μ l transformation pre-culture for inoculation and 1 μ l Golden Gate product from the first part of the workflow. To the 150 μ l transformation culture, 50 μ l of 2% yeast extract is added for regeneration to increase the cell number of the transformed *B. subtilis* cells. This is to increase the probability of selecting transformed bacteria when inoculating the selection culture. The high inoculation volume serves the same purpose. After the selection culture, 1 μ l is used to inoculate a fresh MTP to minimize carryover from the old culture. As a last step in this sub-workflow, cryostocks can be prepared to be stored at −80°C or used to inoculate pre-cultures for cultivation experiments and follow-up assays. For logistical reasons, the transformations were split into three runs as the robotic platform does not have the storage capacity for the number of pipette tips and MTP that would be required to complete the entire experiment in one run.

### 2.4 Automated strain generation

The above mentioned workflow failed to yield the full, rational combinatorial of 28 different amylases with two different promoters and four different signal peptides that transform into 11 different *prsA* background strains. After automated transformation, a quality check was performed by first replicating the liquid-selected strains onto an LB selective plate and a starch plate, which was, in turn, stained using Lugol’s iodine. As can be seen in exemplary Supplementary Figure S10, the transformation of 67 out of 84 strains was successful, and only eight showed amylase activity. Analysis via sequencing of the negative clones revealed that often only one homology arm and the selection marker were integrated (data not shown). As a consequence, a less complex cloning and strain generation approach was chosen. Golden Gate reactions (500 ng total DNA) were set up by combining the Golden Gate fragments for the two different promoters ${\text{P}}_{\mathrm{syn2}}$ and ${\text{P}}_{\mathrm{syn4}}$ , as well as four different signal peptides (YvcE, AmyB, YdjM, and CwlS) with the fragment of one of 19 amylases, and transforming them into 11 different *prsA* backgrounds via the above described workflow. Thus, 19 Golden Gate reactions were used for totally 209 transformations. The resulting clones were transferred to starch agar plates to verify amylase activity and preserved as cryocultures.

### 2.5 Cultivation of generated clones and amylase measurements

Eight individual clones per amylase were inoculated from cryocultures and cultivated in 96-deep well plates. Amylase activities were measured, and the genetic composition of the individual clones was determined using amplicon sequencing, with results elaborated below.

**FIGURE 3:**
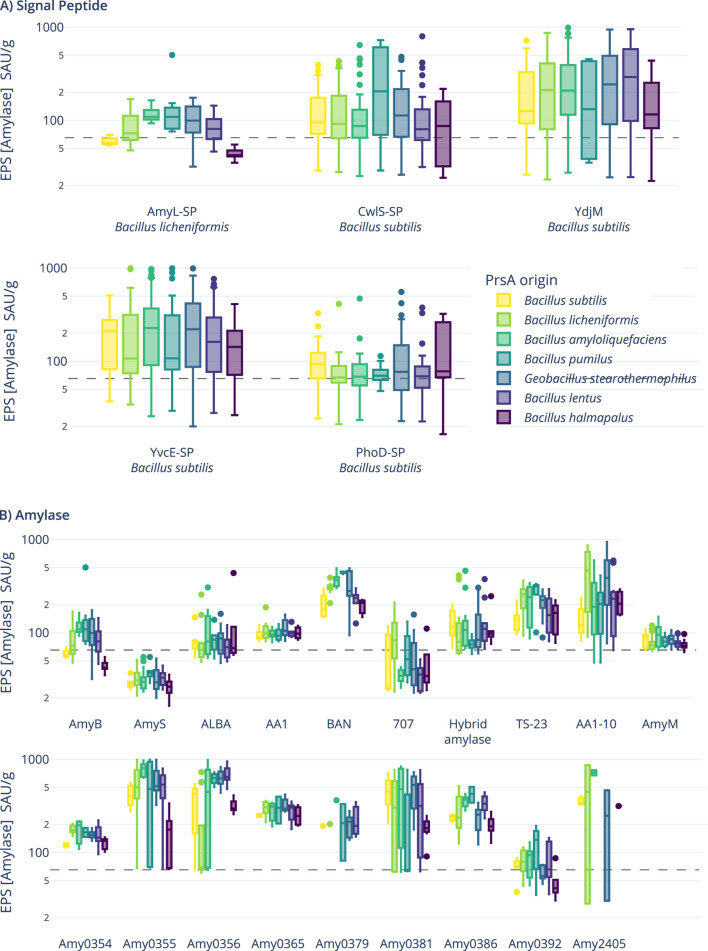
Boxplots of end-point determination of amylase activity (SAU/g: standardized amylase units per Gram). **(A)** Amylase activity plotted per signal peptide resolved by PrsA chaperones present in the strains. **(B)** Activity plotted per used amylase and resolved by the PrsA molecule. The gray dashed lines are the background activities of the negative controls, which, as all strains, contain the native amylase (AmyE) from *B. subtilis*.

As shown in Figure 3A, the signal peptide from YdjM (a putative cell wall hydrolase, BSU_06250), closely followed by the signal peptide from YvcE (D,L-endopeptidase-type autolysin, BSU_34800), outperformed the other signal peptides overall. When resolved by the individual PrsA molecules present in the hosts, the AmyL signal peptide from *B. licheniformis* does not perform best with its native PrsA but instead with the chaperone from *B. amyloliquefaciens*, which also appears to support the secretion of the YdjM and YvcE signal peptides. On the amylase level (see Figure 3B), Amy0355 closely followed by Amy0365 performed best, while AmyS and 707 perform worse than the controls. When viewing the overall performance of the PrsA chaperones (see Supplementary Figure S11, middle), no clear best-suited candidate can be identified, but consistent with previous experiments, PrsA from *B. halmapalus* performs worst.

### 2.6 Amplicon sequencing of cultivated clones

Cultivation was performed in a structured manner, with one MTP cultivation performed for each amylase molecule. This allows, after pooling of respective MTP wells, the use of the amylase sequence as a preset barcode to identify the construct in each clone via PCR amplicon sequencing (Mamanova et al., 2010; Head et al., 2014). Full genotypes of the expression cassette (promoter, signal peptide, and amylase) could be assigned to roughly two-thirds of the clones, while for the remaining clones, only parts of the genotype could be resolved.

## 3 Discussion

Genetic optimization of production strains necessitates the tuning of multiple genetic elements, as demonstrated by previous examples with *B. subtilis* (Westers et al., 2004). The six potential parent strains analyzed at the beginning of this work served as the foundation for such strain optimization. Resistance to lysis was of paramount importance since α -amylases are produced mainly during the post-exponential phase of growth (Gupta and Rao, 2014). Lysis not only reduces product yield (Wang et al., 2014) and purity of the secreted enzymes but also points to low strain fitness. The best growing strains were S19002 and S19006, which reached high optical densities (Figure 1A) and showed reduced lysis (Figure 1C). This is likely due to the modified genotypes (Supplementary Table S1) and natural mutations. S19006 has deletions in the major lysis gene *lytC* (Kabisch et al., 2013), which encodes the major extracellular proteases (Blackman et al., 1998); *xpf*, the sigma factor; as well as *skfA* and *sdpC*, which encode cannibalism factors (Wang et al., 2014). Strain S19002 is more heavily modified with deletions of *lytC*, *skfA*, and *sdpC* but also deletions of *srf*, a surfactin synthase that has been shown to reduce the survival of *Bacillus subtilis* in prolonged cultivations (Tsuge et al., 2001), as well as genes involved in biofilm formation (tas-operon, epsA- and epsO-operons, and surfactin-operon) and toxin–antitoxin systems (endB-ndoA and spoIISA-operon). Based on the LabChip results, the assumption is reasonable to assume that the decrease in ${\text{OD}}_{600}$ after the initial peak in the other strains is caused by *B. subtilis*. The multi-protease knock-out S19006 was used in the initial research up to automatic cloning because amylases are susceptible to protease degradation (Stephenson and Harwood, 1998), which is required for a platform screening strain. In parallel, protease knockouts in S19002 were pursued, resulting in strain S19034 with a total of 19 deletions. In order to ensure that 29 uses of a site-specific recombinase do not result in genome rearrangements, S19034 was sequenced, confirming the expected genome structure and demonstrating the power of the utilized Cre-Lox system for marker recycling. In the genotype, some additional frameshifts in contrast to the original sequencing by Kabisch et al. (2013) could be revealed.

It is established from the literature that the overexpression of PrsA (Vitikainen et al., 2001) and choosing a different PrsA (Quesada-Ganuza et al., 2019) can be beneficial for amylase secretion. The investigation of effects that different additional *prsA* genes have on the production of recombinant amylases in this study showed that PrsA in most cases has a positive effect on amylase activity (Supplementary Figure S5). This observation is consistent with the results obtained by Quesada-Ganuza et al. (2019), which increased amylase production by optimizing PrsA chaperones. However, it is worth mentioning that all strains still had the native *B. subtilis prsA* gene. It was expected that introducing an additional copy of a *prsA* gene would increase the amylase production and secretion since that is the case even for the overexpression of the native chaperone (Chen et al., 2015; Kontinen and Sarvas, 1993; Vitikainen et al., 2005). *prsA* 457 from *B. halmapalus* was the only one that had a negative effect on amylase activity in both *amyB* and *amyS* strains (Supplementary Figure S5). This implies an inhibitory function in either secretion or folding specific to the tested amylases, as well as a negative effect on the host caused by an unsuitable PrsA. Further research would be required to confirm this theory. *prsA* 457 was nevertheless continued to be examined in the following experiment as a negatively influencing gene. In addition to the chaperone gene, the promoter driving *prsA* expression appeared to have an impact on the growth of the strains, with promoter 4 showing the highest ${\text{OD}}_{600}$ after 72 h in both *amyS* and *amyB* strains (Supplementary Figure S4). Looking at the growth of strains compared to protein production, expression-induced stress is often detrimental to growth (Nordholt et al., 2017). This might be one such case. In terms of increasing amylase production, promoter 4 does not produce as promising results as the other promoters in *amyB* strains.

The efficacy of PrsA appears to be highly expression- and amylase-dependent because the activity of cultures transformed with the same PrsA varied significantly depending on the used promoter and amylase. Hence, there was no single PrsA that could be selected as the best option for all or most amylases and/or promoters. The diversity of activities in different amylase–PrsA promoter combinations is striking and indicates that balancing all components might be the key to success.

Efficient screening requires parallelized cultivation preferably in MTP plates. Several issues stemming from cultivation in the Cytomat2 MTP shaker had to be identified and addressed. One of these was plate abrasion and increased evaporation on the plate edges caused by insufficient fixation of the MTP lids during shaking, which was solved through an engineering student project (see Supplementary Figure S12). MTP results generally had more outliers than DWP results. With an evaporation test (Supplementary Figure S6), one of the causes was confirmed to be positional effects in the MTP incubator. The shape of ${\text{OD}}_{600}$ increase, which can be observed in the outliers of previous MTP cultivations (Supplementary Figure S4), was most probably caused by evaporation since it only occurred on the edges of plates and was supported by visible changes in liquid levels. Position 13 is closest to the incubator door and, therefore, had the highest ${\text{OD}}_{600}$ of all positions. The outliers in P14 rows A–C might have been caused by the fans located directly beneath that area of the MTP. Evaporation is a problem that needs to be considered because it can produce a snowball effect when the higher surface to volume level leads to higher ${\text{O}}_{2}$ saturation (Wittmann et al., 2003), which leads to faster growth, and in turn, media components might change with osmolarity increases drastically. Evaporation is also especially severe in MTP because of small volumes, as suggested in Betts and Baganz (2006). It is thus important to determine evaporation effects in MTP for each automation setup. The DWP cultivations, on the other hand, produced much more consistent results because of the decreased risk of losing too much volume during longer cultivation (Sieben et al., 2016). Although breathable membranes inhibit oxygen transfer (Zimmermann et al., 2003), the square shape of plates creates turbulence inside the culture, which increases oxygenation together with the higher throw in the incubator (Duetz et al., 2000). As detailed in Bareither and Pollard (2011), several laboratories have proven that DWP can also produce a predictive scale for stirred tanks up to 10 L.

A workflow has been devised for rapid cloning and the generation of many different amylase-integrated strains in one run. It was planned to, all in all, create 2,240 strains, which should be cultivated in MTP and DWP and screened for their amylase activity. The cloning and cultivation process was split into three functional, automated modules, each with a save point that enables pausing the process without losing progress. Designing automation processes in modules is extremely useful as it makes the automation process less complex and, therefore, less prone to errors. Such errors are mostly caused by the automated handling of labware, such as stuck lids, tips falling off, robot arm dropping labware, or missing labware because the operator forgot to load it or misplaced it. Furthermore, once a module has been well established, it can be reused for other processes.

Transformation cassettes were cloned via Golden Gate (Engler et al., 2008; 2009) reactions. The contactless nanoliter dispenser (I.DOT, Dispendix) enabled high-frequency, low volume dispensing of Golden Gate parts, making these types of devices essential for automation setups working with DNA assemblies (Holowko et al., 2021). The transformation of the obtained Golden Gate constructs into the 10 *B. subtilis* strains containing different *prsA*–promoter combinations was established to be fully automated by using a liquid selection process instead of plating and colony picking, which is much harder to implement as work moves away the structured plate/well format. The developed transformation method in MTP has been tested successfully in experiments conducted manually. Since we did not include colony PCR and our testing relied on starch-based amylase plate assays, the method depends on the reliability of the Golden Gate reaction and transformation. Engler et al. (2009) reported an efficiency of 97% for nine different modules in *E. coli*, while the aim of this work is just combining three of them. The amylase assay proved to be sufficient to detect that a full combinatorial DNA assembly generating 2,240 strains was not feasible without substantial optimization. For the presented workflow, manual picking was a task readily performed within 1 hour including replica plating using a 96-spike picker. The automated transformation worked as we received spectinomycin-resistant colonies that were negative for native amylase activity (derived from AmyE), indicating successful uptake of DNA and integration into the target locus; however, higher transformation rates might have solved the problem of many clones integrating only the antibiotic resistance gene and one homology arm. Fixing the frame shift in *comP*, which is involved in the development of natural competence (Maier, 2020), might have provided that increase. The fact that the cells can still become competent indicates that the point mutations that cause early stop codons are to some degree suppressed (Belinky et al., 2021). In order to overcome this construct generation bottleneck, we opted to proceed with a reduced combinatorial approach of one-pot reactions for the 19 amylase genes. In this study, the fragment for the signal peptide and the promoter was shuffled with a single amylase gene and transformed in a defined PrsA strain. The genotypes were assigned to the individual clones via amplicon sequencing of pooled clones, with the known amylase representing the barcode. This resolved the full genotype in two-thirds of the cases. For one-third of clones, only parts of the genotype could be determined due to the application of short-read sequencing with short average fragment sizes of the sequencing libraries, which did not span all construct element boundaries. For future approaches, long-read sequencing could be used to improve the identification of genotypes (Currin et al., 2019).

The screening results for optimal PrsA–amylase combinations can be easily summarized: no single, optimal PrsA molecule that always improved amylase production could be identified; however, within a group, different combinations of genetic elements and PrsA molecules could lead to a 10-fold variation in amylase production. For the screened genetic constructs and strains, PrsA from *B. halmapalus*, which is consistent with the results from the pre-experiments, tended to perform worst. Interestingly, growth was not affected, so one might exclude a negative effect on cellular functions such as the folding of penicillin-binding protein 2B (PBP2B), an essential protein for cell wall synthesis depending on PrsA for correct folding (Hyyryläinen et al., 2010). Sequence analysis revealed that the NC domain of the PrsA of *B. halmapalus* is strongly negatively charged with an overall charge of −17 at pH 7. This is consistent with the NC domain of, for example, the PrsA of *G. stearothermophilus* with a net charge of only −2 at pH 7. In a noteworthy publication, Quesada-Ganuza et al. (2019) created modifications in *B. lentus* PrsA associated with reduced hydrophobicity with an improved effect on secretion. The negative impact of *B. halmapalus* PrsA emphasizes the importance of overall charge, at least for the class of amylases. Evidently, further work such as testing hybrids might increase the efficiency of this foldase class to their client amylases.

For the signal peptides, YdjM and YvcE (synonym CwlO) performed best. YdjM was previously identified to be a well-performing signal peptide in protease secretion screening (Degering et al., 2010), and both sequences (YdjM and YvcE) are described to be suitable but not outperforming signal peptides for an alkaline xylanase (Zhang et al., 2016). Recent studies have not reached a clear conclusion about which sequence parameter is essential (Grasso et al., 2023; Freudl, 2018; Zhang et al., 2020; Brockmeier et al., 2006), and no overarching canonical signal peptide is known. As the signal peptide might not only present a marker within the cellular environment for secretion but may also affect mRNA stability, folding of the cognate protein, and cleavage of the pre-protein, more studies, such as by Grasso et al. (2023), are required to shed light on the requirements for efficient, predictable heterologous of secretion.

## 4 Conclusion

The presented automation setup allowed us to automatically generate and transform *B. subtilis* strain-producing amylases in various genetic contexts. Toward this goal, the genome-reduced production strain S19034 was generated with many favorable traits such as the absence of major extracellular proteases and robust growth with strongly reduced lysis. Further shortcomings on cultivation in tower shakers in automation setups were identified and addressed. Advantages of automated strain construction include higher throughput, avoidance of human errors while handling many samples, and reproducible data. Challenges include a high effort for the verification of constructs and high time requirements to develop robust workflows.

## 5 Methods

### 5.1 Strains and cultivation

#### 5.1.1 General cultivation

All *B. subtilis* strains are listed in Supplementary Table S1 with their respective relevant genotypes. Parent strains S19001, S19002, and S19003 were kindly provided by BASF, while S19004, S19005, and S19006 were derived from the strain 6051HGW (Kabisch et al., 2013) and constructed previously in the Kabisch-lab. Cells were cultivated in Luria-Bertani medium with either 10 g/L (LB, Carl Roth, X968.3) or 5 g/L NaCl (LB5, Sigma-Aldrich, L3022-1 KG), when Zeocin was applied with appropriate antibiotics (20 μ g/mL of Zeocin, InvivoGen, ant-zn-1p and 100 μ g/mL of spectinomycin, Sigma-Aldrich, S4014-25G) at 37°C 200 rpm (INFORS HT Multitron II, 25 mm throw). *E. coli NEB 10* β was used for plasmid propagation and cloning purposes. Liquid cultures were grown in culture tubes at 37°C at 200 rpm in LB medium supplemented with 100 μ g/mL of spectinomycin when appropriate. Cryostocks were created by adding 20% of glycerol to bacteria cultures and freezing them at −80°C.

#### 5.1.2 Cultivation experiments

Cultivation experiments were carried out at 30°C using MTP (Greiner, 96-well plate, clear, flat bottom, M4811-40 EA) incubated in a Cytomat2 incubator (Thermo Fisher Scientific, US-MA) with 1,000 rpm (amplitude 1.5 mm) and plastic lids (Greiner, 656101). DWPs (Whatman, 96 deep-well plates, round bottom, 734–2,559) were incubated at 300 rpm (New Brunswick Innova 44, 51 mm orbit) and covered with adhesive gas permeable seals (Thermo Scientific, AB0718) in previously described 3D printed cultivation boxes (Bruder et al., 2019). A measure of 600 μ l of Terrific Broth (TB) medium (10 g/L glycerol, 12 g/L tryptone, 24 g/L Yeast extract, 12.54 g/L ${\text{K}}_{2}{\text{HPO}}_{4}$ , and 2.31 g/L ${\text{KH}}_{2}{\text{PO}}_{4}$ ) pre-cultures were inoculated either from the agar plate or cryostock with one colony per well and cultivated in DWP overnight. A mineral salt medium was used for the main cultures in microtiter plates. The mineral salts and trace elements contained in the mineral salt medium are described in WO/2020/169,564 or Habicher et al. (2020). The mineral salt medium was supplemented with C-source, as described by Tännler et al. (2008), and adjusted to pH 7.6 using 5 M NaOH , followed by sterile filtering with 0.2- μ m filters. Although DWP were only incubated for end-point measurements, growth in MTP was tracked via automated ${\text{OD}}_{600}$ (BMG Labtech PHERAstar FSX) measurements every 1 h (see Automation). The main culture cultivation in both plate types was stopped after 72 h, manual end ${\text{OD}}_{600}$ measurement was taken at a 1:20 dilution (BMG Labtech CLARIOstar Plus), and the plates were stored at −20°C.

### 5.2 Cloning

#### 5.2.1 DNA manipulations

Polymerase chain reaction (PCR) was performed as described in Kuslich et al. (2019), with hybrid polymerase (Roboklon, E2950-02) relying on the polymerase manual for annealing temperature calculation. Oligonucleotides were obtained from Sigma-Aldrich (Supplementary Table S4). Colony PCR was conducted according to Woodman et al.’s (2016) protocol, with an initial denaturation time of 10 min using either Taq (Roboklon, E2600-02) or OptiTaq polymerase (Roboklon, E2600-02). Agarose gel electrophoresis was performed as described in Armstrong and Schulz (2015), with ROTI GelStain (Carl Roth, 3,865.1) and 1 kbp DNA ladder (Carl Roth, Y014.2) or 1 kb Plus DNA ladder (New England Biolabs, N3200L) for DNA quantification. Genomic DNA was extracted using the High Pure PCR Template Preparation Kit (Roche, 11796828001). The innuPREP Plasmid Mini Kit 2.0 (Analytik Jena, 845-KS-5041250) was used for plasmid preparation and NucleoSpin Gel and PCR Clean-up kit (MACHEREY-NAGEL, 740609.50) for PCR cleanup. All DNA extractions were followed by NanoDrop (Mettler Toledo, UV5Nano) concentration measurements and an agarose gel. DNA was sequenced by Eurofins Genomics using the Mix2Seq Service.

#### 5.2.2 Cloning of amylase and *prsA* expression cassettes

The Golden Gate protocol was adapted from Engler et al. (2008) and Engler et al. (2009), with the reaction mix containing 200 ng of each Golden Gate part: 1.5 μ l of 10x buffer G (Thermo Scientific, BG5), 1.5 μ l of BSA (1 mg/mL in Milli-Q, New England Biolabs, B9000S), 0.75 μ l of adenosine triphosphate (ATP, Carl Roth, K035.1), 1 μ l BpiI (Thermo Scientific, ER1012), and 1 μ l of T4 DNA Ligase HC (Promega, M1794) filled up to 15 μ l of Milli-Q water (Sigma-Aldrich) in PCR tubes (Sarstedt, 72.737.002). ATP and ligase were added after the sample was incubated at 37°C for 2 h as a pre-digestion step. The digestion–ligation reaction was done in 35 cycles of 10 min 37°C and 5 min 16°C, followed by 5 min at 50°C and 5 min heat inactivation at 80°C (DNA Tetrad 2 Peltier Thermal Cycler, Bio-Rad).

#### 5.2.3 Cloning of deletion plasmids using SLiCE

Deletion plasmids were constructed using SLiCE, according to Zhang et al. (2012). Homology regions were amplified from isolated genomic DNA of S19002 via PCR using hybrid polymerase. The SpecR cassette with lox and six sites [*lox-SSS* (Kumpfmüller et al., 2013)] was amplified in two parts from the chromosomal DNA of strain BsFLN040, a pJet backbone which was obtained via amplification from plasmid DNA p19012. After DNA cleanup of all components (New England Biolabs Sequencing Monarch PCR and DNA Clean Up), the SLiCE reaction was started. Electrocompetent *E. coli NEB 10* β was transformed with the whole SLiCE mix via electroporation, according to the protocol obtained from Morrison (1997) with 450 μ l of LB instead of SOC for regeneration. After the confirmation of the correct plasmid assembly via colony PCR and sequencing (Eurofins genomics Mix2Seq), successfully transformed strains were cultivated for plasmid extraction and cryostock creation.

#### 5.2.4 *Bacillus subtilis* strain generation

Transformations of *B. subtilis* were accomplished by adding 50 μ l of overnight culture to the transformation broth (13.7 mg/mL of ${\text{K}}_{2}{\text{HPO}}_{4}$ , 6 mg/mL of ${\text{KH}}_{2}{\text{PO}}_{4}$ , 1.68 mg/mL of trisodium citrate, 2% (w/v) of D-glucose, 11 mg/L of ammonium iron (III) citrate, 0.2% (w/v) of potassium glutamate, and 3 mM of ${\text{MgSO}}_{4}$ ) supplemented with 20 μ l 5% (w/v) of Casein hydrolysate to 950 μ l of the same mixture, together with 500–1,000 ng of DNA. After 5–6 h incubation at 37°C at 200 rpm, the entire culture was plated on LB agar supplemented with appropriate antibiotics. Transformation was confirmed via colony PCR with Taq polymerase ( ${\text{EUR}}_{x}$ ), followed by 1% agarose gel electrophoresis. All permanent changes to strains were also checked with the sequencing (Eurofins Genomics, Mix2Seq) of PCR fragments obtained via colony PCR with OptiTaq and subsequent PCR purification (MACHEREY-NAGEL, 740609.50). In S19002, Cre-Lox recombination was performed to heal spectinomycin resistance, which is formerly integrated as a selection marker by using the built-in *Cre* recombinase. The expression of the recombinase was induced by cultivating in ventilated 2-mL microcentrifuge tubes (Sarstedt, 72.695.500) with 300 μ l of LB and 1% (w/v) xylose at 37°C at 200 rpm for 6 h. The culture was then streaked on LB agar to obtain single colonies, which could again be picked on LB agar and LB Spec100 agar plates. Colonies which did not grow on LB Spec100 agar were tested for successful removal of the resistance via colony PCR and sequencing. S19006 was transformed transiently using a plasmid containing the *Cre* recombinase and a heat-sensitive origin of replication derived from Altenbuchner (2016) (p11082_M) since it has no integrated *Cre* recombinase. Transformed cells were plated on LB agar plates using 50 μ g/mL of kanamycin (LB Kan) and 1% xylose for the induction of *Cre* recombinase expression. Plates were incubated at 30°C overnight and streaked on LB agar plates, which were incubated at 50°C to erase the plasmid from the resulting colonies. These were then picked onto an LB agar plate, an LB Kan50 plate, and an LB Spec100 plate. Colony PCR was then performed on the colonies, which did not grow on the antibiotics plates.

#### 5.2.5 Downscaling of *B. subtilis* transformation to microplates

The Golden Gate reaction and its PCR-amplified products were run on a 1% agarose gel and quantified using a DNA marker and free GelAnalyzer software to measure the amount of the relevant construct in each sample and calculate the amount of DNA required for a scaled-down transformation mix. The concentrations of the relevant bands were 40 ng/ μ l in the Golden Gate mix and 90 ng/ μ l in the PCR amplification. When scaled to 150 μ l, 0.75 μ l of the PCR was used with 1.5 μ l of the Golden Gate product. Six wells were used per transformation assay with six controls containing no transformation DNA. Each well was inoculated with 1.5 μ l of the S19034 overnight culture in the transformation mix. One part was regenerated by adding 7.5 μ l of 10% yeast extract, and the other was regenerated as well, with the addition of spectinomycin to a concentration of 200 ng/ μ l. After a regeneration phase of 2 h, 100 μ l of the transformation culture was taken to inoculate an LB5 culture with a total volume of 200 μ l, with 200 ng/ μ l of spectinomycin and 20 ng/ μ l of Zeocin. This MTP was then incubated at 37°C overnight. From this culture, 1 μ l was used to inoculate 200 μ l of LB5 Spec200 Zeo20 in a new MTP.

### 5.3 α -Amylase activity assays

Amylase activity was measured with an assay described by Lorentz (2000). For the calibration curve, a dilution series of 1:2 starting at 1:16 and ending at 1:512 of the amylase standard (Termamyl 120L, Sigma 3,403 Lot: SLBJ0544V with a given concentration of 18400 SAU/g; SAU/g = sigma amylase units/g) in MOPS buffer (50 mM of 3-morpholinopropane-1-sulfonic acid, 50 mM of NaCL, and 3 mM of ${\text{CaCl}}_{2}$ dissolved in desalinated ${\text{H}}_{2}\text{O}$ and pH adjusted to 7.1 with NaOH) diluted at a ratio of 1:10 (=55.2 SAU/g) was made and measured as follows: 100 μ l of MOPS buffer was aliquoted in each well of an MTP. A measure of 100 μ l of the culture supernatant was added to row A of the MTP and then diluted in 1:2 steps to row H. The start solution was prepared by mixing the glucosidase solution (56.8 U/mL of α -glucosidase multifunctional, Roche Diagnostics Lot. 13866247, in MOPS buffer) at a ratio of 1:1 with the EPS stock solution (14 mM of ethyliden-4-NP-G7, Roche Diagnostics; 1,300.1 g/mol in MOPS buffer). A measure of 100 μ l of the start solution was added to each well, and fluorescence at 405 nm was recorded immediately (Molecular Devices SpectraMax M3) for 600 s, reading every 30 s. During the assay, a temperature of 30°C was maintained. SoftMaxPro 5.4.4 was used for calculating the standard curve using linear regression. Subsequently, activities of the samples were calculated in SAU/mL.

### 5.4 Automation

Automation was applied to several degrees. Generation of cryostocks, for example, was significantly more robust when using a semi-automated 96-well pipette (CyBio SELMA, Analytik Jena) as the operator had visible control over potential cross-contamination, such as bubbles at the tip-ends popping and spreading cells through various wells. All automation processes in the robotic platform were designed with as few steps as necessary to reach a stop and back-up point (see Figure 2. Pipetting of the Golden Gate reaction, for example, was done manually loading the nanoliter dispenser (I-Dot, Dispendix) with source and target plates. After successful liquid transfer, the plates were manually transferred to a thermocycler for the thermal reaction, which proved to be faster and more robust than starting from several hotel positions and using the built-in thermocycler for the required throughput. To characterize the growth of the strains, an automated setup using a microplate tower shaker with controlled humidity (Cytomat2, Thermo Fisher Scientific) was used for incubation. For OD measurements, plates were transferred between the Cytomat and a plate reader (BMG Labtech PHERAstar FSX) using a robotic arm (PreciseFlex 750, Brooks Automation). A representation of the complete robotic platform without the housing is shown in Supplementary Figure S1. Because the MTP cultivation used by Cytomat2 was prone to massive abrasion from the plastic lids, the tower shaker was modified by attaching 2-mm ethylene–propylene–diene cellular rubber to the back of the holders using superglue (see Supplementary Figure S12).

### 5.5 Amplicon sequencing of pooled MTP cultivations

For sequencing, the MTP plates were re-grown from a backup glycerol stock in LB media supplemented by 20 ng/ μ l of Zeocin. From each plate, identical wells were pooled (MTP 1 well A1 + MTP 2 well A1 + MTP 3 A1, etc.), and total DNA of each pool was isolated via EtNa DNA extraction (Vingataramin and Frost, 2015). From the total DNA, PCR amplification was performed using primers 150 bp upstream of the promoter sequence within the amyE gene (FEM3868; 5′-AGA​TGA​TGG​CAG​TTA​CGG​CAG-3′) and 50 bp downstream of the amylase sequence (FEM3868; 5′-ATA​AGG​CCG​CCT​CTT​AAC​GG-3′). The PCR fragments had a size of approximately 2.5 kB (exact size dependents on the inserted amylase molecule), covering the promoter–signal peptide and amylase sequences. From these PCR products, sequencing libraries were generated using the NEBNext^®^ Ultra™ II DNA Library Prep Kit for Illumina^®^ (E7645, New England Biolabs) automated on a Biomek i7 Workstation (Beckman Coulter Life Sciences). Sequencing was performed on an Illumina MiniSeq System (Illumina). The resulting sequence fragments were assembled, and using the amylase sequence as the barcode, the expression cassettes could be annotated to each cultivation plate.

### 5.6 Data analysis

Data were analyzed using RStudio 3.6 with R version 3.6.1 on x86_64-pc-linux-gnu (64-bit) running under Ubuntu 19.10 with the following packages: growthcurver 0.3.0, viridis 0.5.1, viridisLite 0.3.0, gridExtra 2.3, cowplot 1.0.0, forcats 0.4.0, stringr 1.4.0, purrr 0.3.3, readr 1.3.1, tidyr 1.0.2, tibble 2.1.3, ggplot2 3.2.1, tidyverse 1.3.0, dplyr 0.8.3, and magrittr 1.5. Interactive plots were generated using plotly 5.18.0 and pandas 2.1.3. Geneious 11.1.5 was used for genetics. GelAnalyzer 19.1 was used for the gel analysis of DNA concentrations. For the calculation of significance, a Shapiro–Wilk test for normal distribution was done before two-way ANOVA, and then, Tukey’s honest significant difference test was performed.

### 5.7 LabChip measurements

Measurements were conducted with a PerkinElmer LabChip device according to manufacturer’s instructions using the Protein Express Assay Reagent Kit (PerkinElmer, CLS960008) with an AA560 amylase variant (55 kDa, 483aa) as a reference.

## Data Availability

The datasets presented in this article are not readily available because sequence data for proprietary sequences cannot be provided. Requests to access the datasets should be directed to johannes.kabisch@ntnu.no.
