# Supplementary material for: Automation-aided construction and characterization of Bacillus subtilis PrsA strains for the secretion of amylases
Source: Front Bioeng Biotechnol. 2025 Jan 23;12:1479626. doi: 10.3389/fbioe.2024.1479626 (PMC11798935; doi:10.3389/fbioe.2024.1479626)
Supplement: Supplementary file 1 [file DataSheet1.pdf]

Automation-aided construction and characterization of *Bacillus subtilis* PrsA strains strains for the secretion of amylases: supplemental document

December 8, 2024

Contents

1 Introduction **S1**

2 Supplemental Figures **S1**

3 Supplemental Tables **S10**

1 Introduction

This supplemental document contains figures referenced in the main manuscript as well as additional material and methods.

2 Supplemental Figures

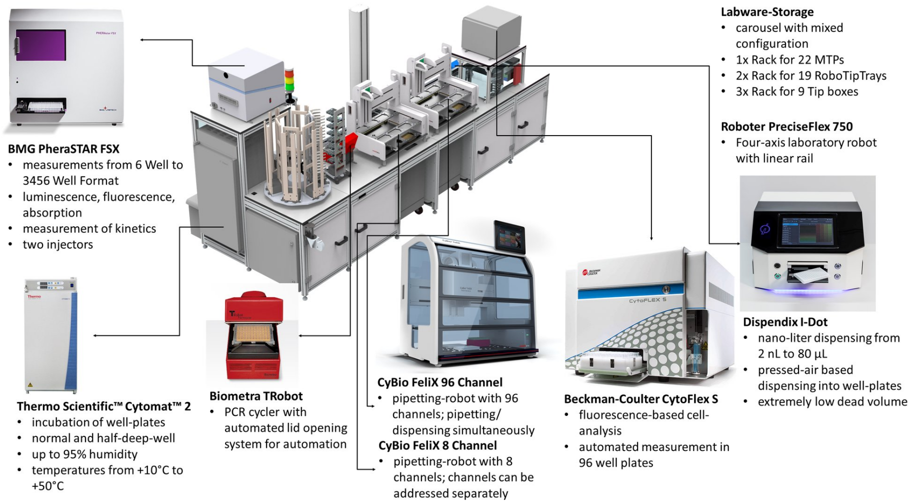

Figure S 1: Rendering of the utilized robotic platform constructed by Analytik Jena AG including description of its major components. Not labelled: Precise-Flex 750 robotic arm, labware hotel and various labware holding positions and a de-lidding station.

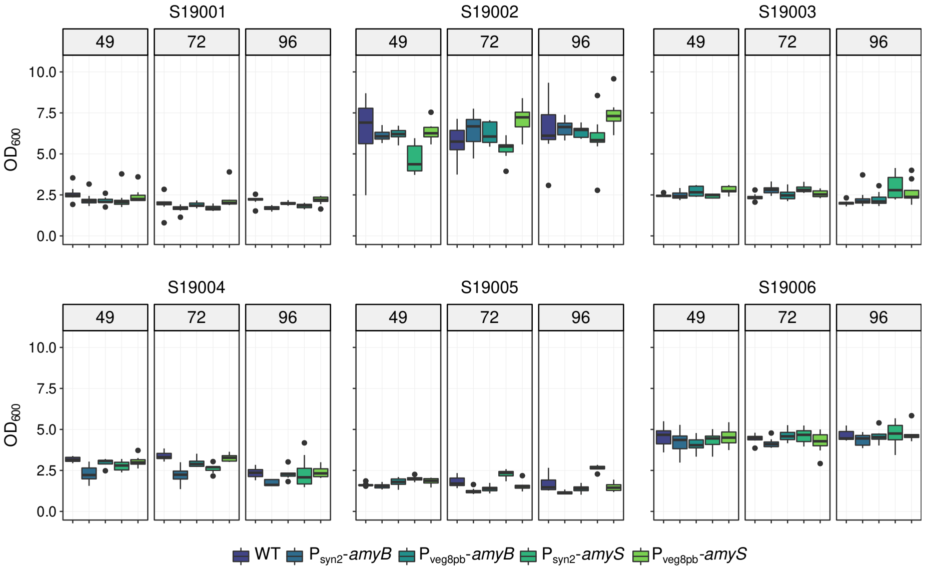

Figure S 2: Endpoint OD_{600} measurements of all recorded incubation times. Strains were transformed with amylase cassette PCR products. PCR1:P$_{s}yn2$-amyB, PCR2: PP$_{v}eg8pb$ -amyB, PCR3: P$_{s}yn2$ -amyS, PCR4: PP$_{v}eg8pb$ -amyS. Cultivation in deepwell plates at 30°C with 300 rpm. OD_{600} was measured in 1:20 dilution in a microplate reader after 49, 72 and 96 h.

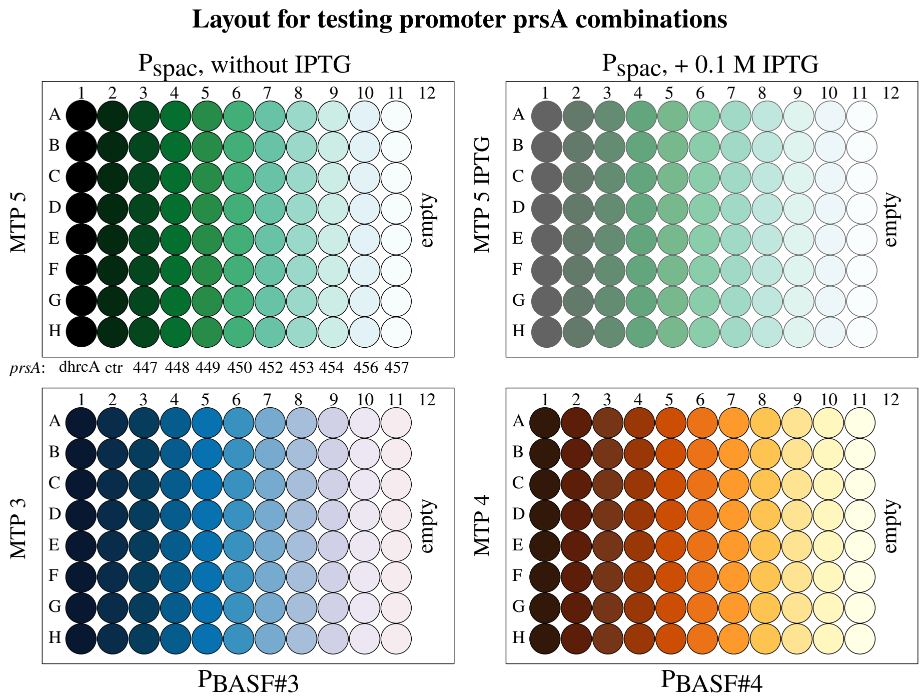

Figure S 3: Layout of the cultivations for testing *prsA* genes with different promoters. Numbers refer to the PrsA listed in Table S2. dhrcA: hrcA-deletion mutant, this data is not shown nor discussed; ctr: native *B. subtilis* amylase (AmyE)

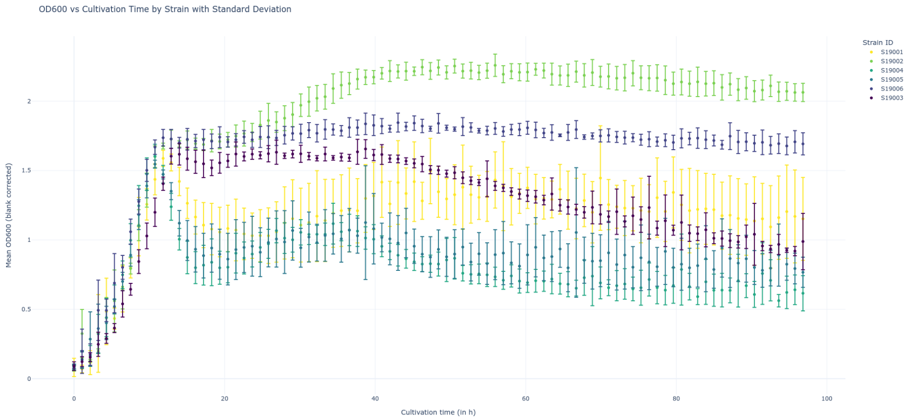

Figure S 4: Alternative plot of Figure 1a from the manuscript showing all growth data in one plot.

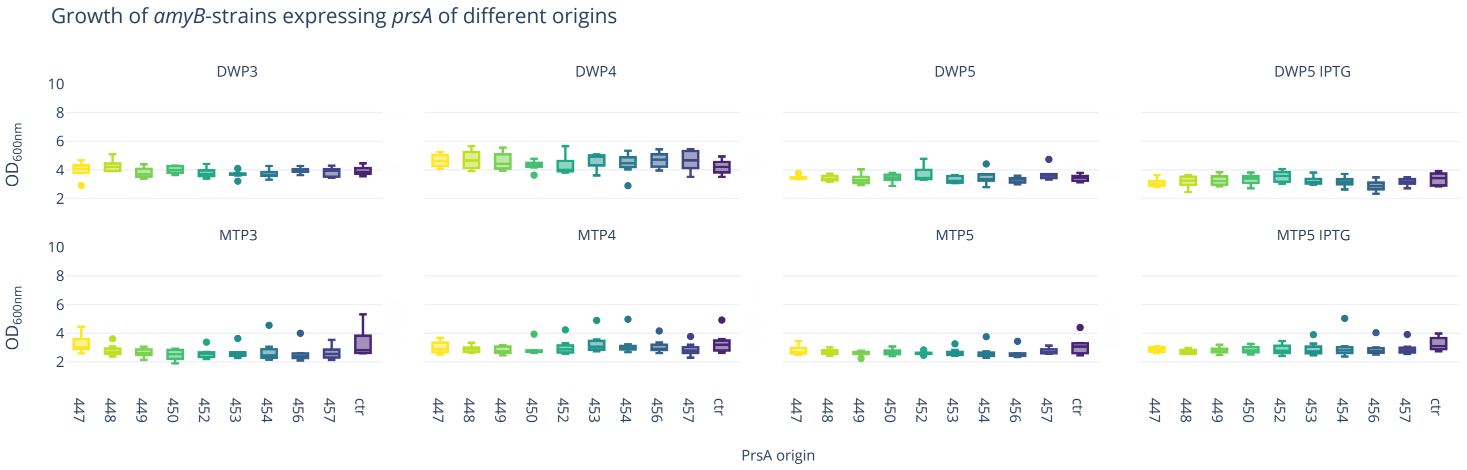

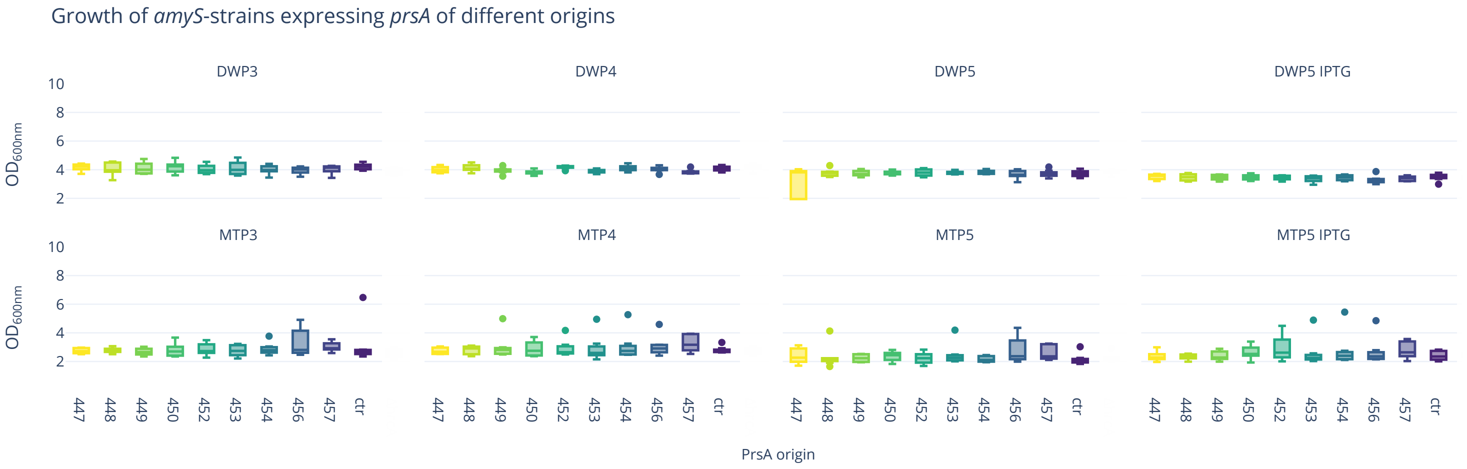

Figure S 5: End-point cultivation OD data of *prsA*-transformed *amyB* strains and *amyS*-strains, split by promoter indicated as number and MTP/DWP. a) S19006 *amyB* was transformed with *prsA*-cassettes. For each *prsA*, eight different transformed colonies were picked and cultivated for 72 h in either the robotic platform at 30 °C 1000 rpm (MTP with plastic lid) or 30 °C 300 rpm (DWP with gas permeable seals). OD_{600} was measured afterwards in a 1:20 dilution on a microplate reader. b) The supernatant of the same *amyB* culture plates was then used for amylase activity assays, the results of which are shown here. With a sample number of $n = 8$, the mean activity was calculated in SAU/ml (Sigma Amylase Units / ml). Error bars show standard deviation. Numbers on the x-axis refer to the PrsA-molecules listed in Table S2.

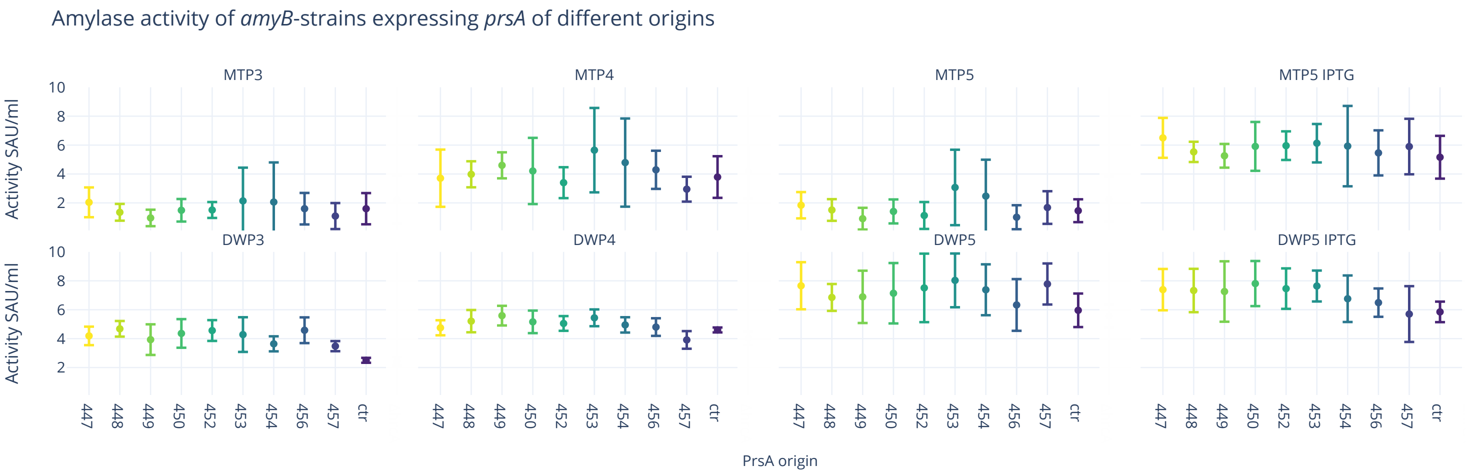

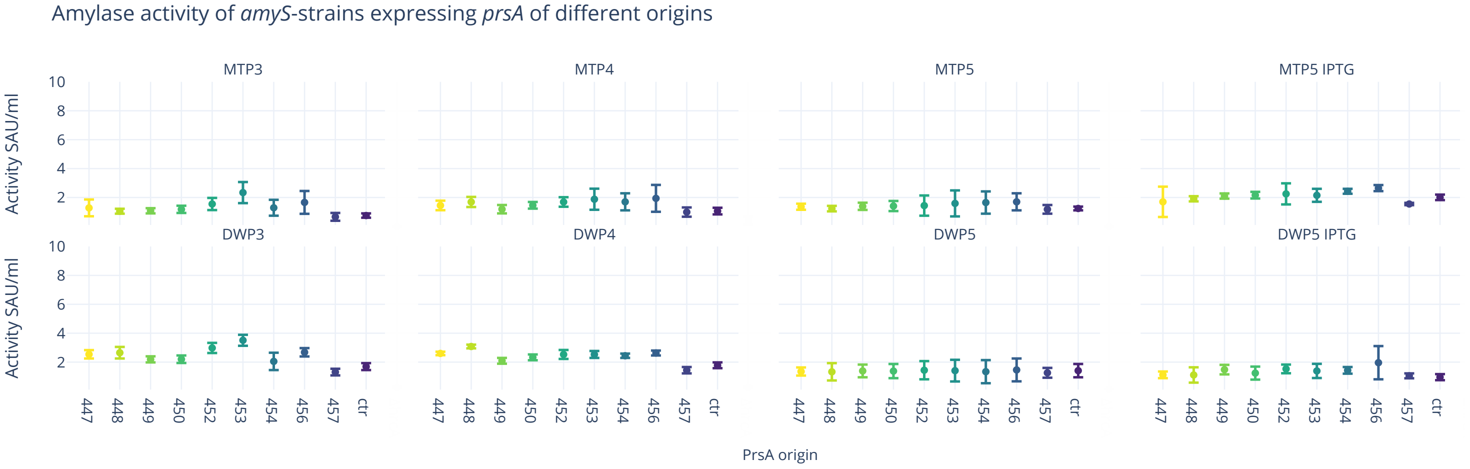

Figure S 6: Activity data of *prsA*-transformed *amyB* and *amyS* strains, split by promoter indicated as number and MTP/DWP.a) S19006 *amyS* was transformed with *prsA*-cassettes. For each *prsA*, eight different transformed colonies were picked and cultivated for 72 h in either the robotic platform at 30 °C 1000 rpm (MTP with plastic lid) or 30 °C 300 rpm (DWP with gas permeable seals). OD_{600} was measured afterwards in a 1:20 dilution on a microplate reader. b) The supernatant of the same *amyS* culture plates was then used for amylase activity assays, the results of which are shown here. With a sample number of $n = 8$, the mean activity was calculated in SAU/ml (Sigma Amylase Units / ml). Error bars show standard deviation. Numbers on the x-axis refer to the PrsA-molecules listed in Table S2.

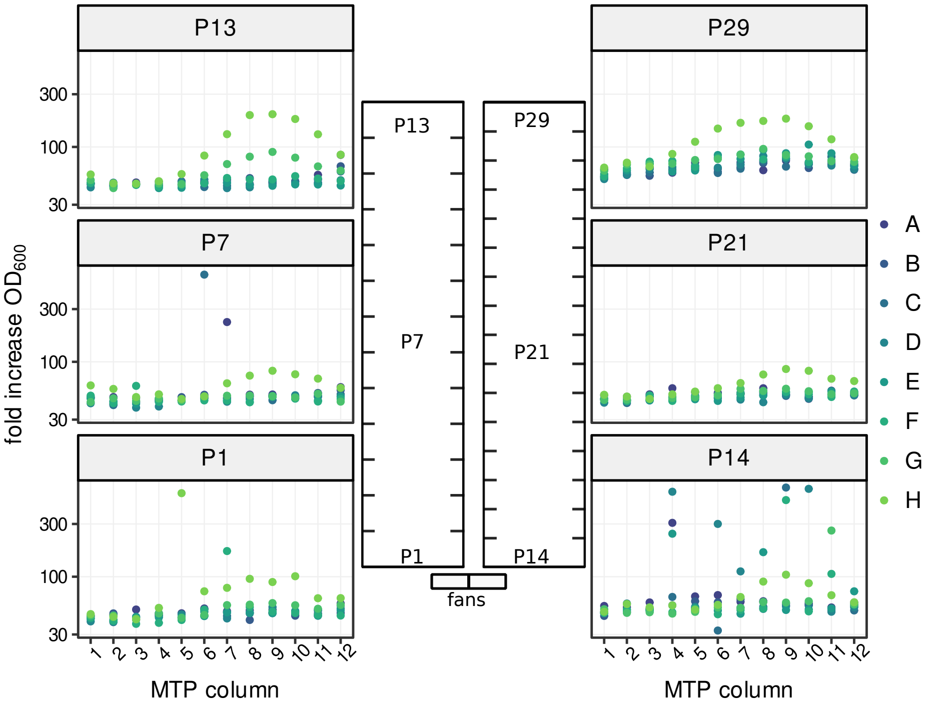

Figure S 7: Positional effects test for cultivation in the robotic platform. Main culture medium was inoculated 1:100 with S19006 preculture and then distributed into MTP for cultivation in the robotic platform. OD_{600} was measured in a microplate reader before cultivation and after 72 h of cultivation at 30 °C 1000 rpm in a dilution of 1:20. From the disparity between the start- and end value of each well, the fold increase in OD_{600} from start to finish was calculated. This value is shown here. Rows of MTP are color coded, columns are shown on the x-axis. MTP were placed in six different positions of the shaking towers in the incubator as illustrated in the graphic.

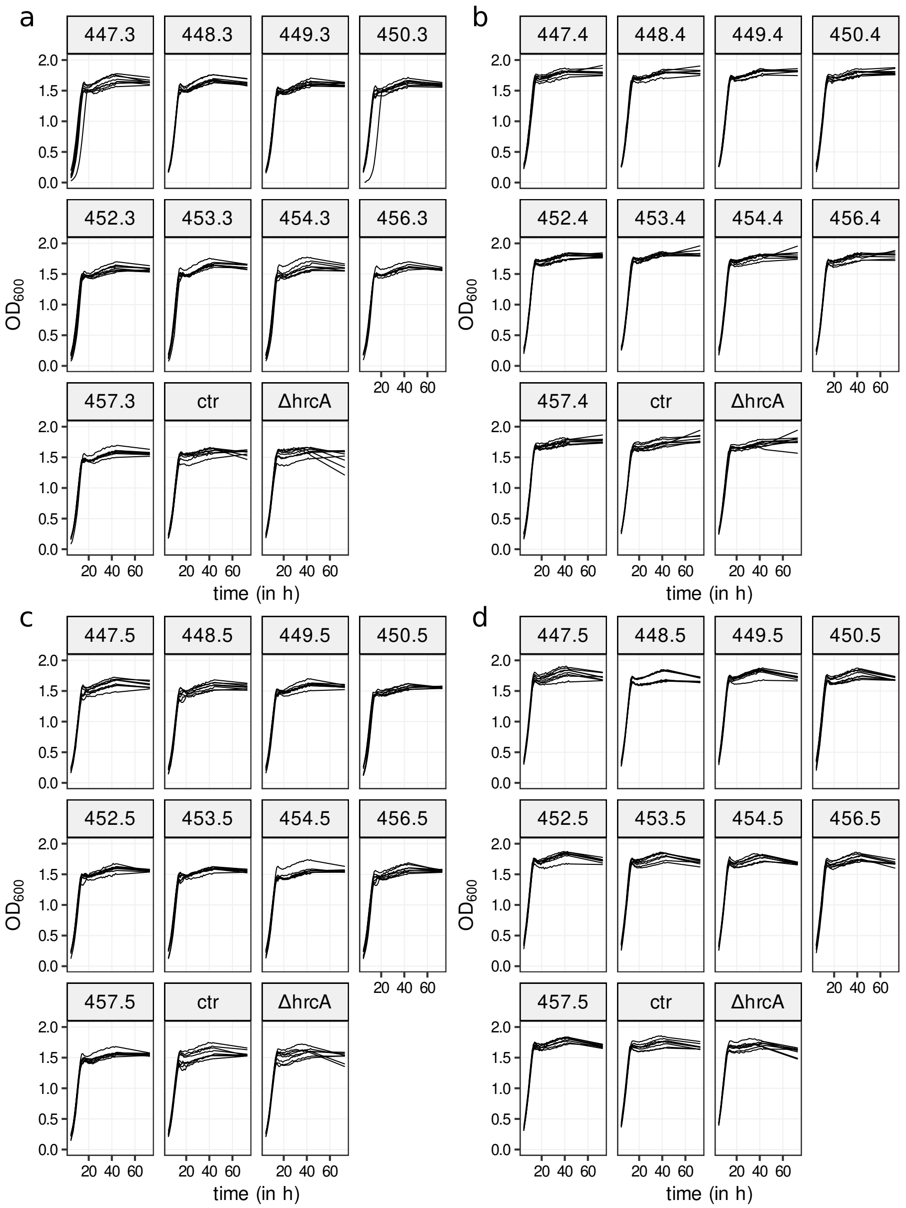

Figure S 8: All growth curve data gathered during cultivation of PrsA test in *amyB* strains. S19006 *amyB* was transformed with *prsA*-cassettes. For each *prsA*, eight different transformed colonys were picked and cultivated for 72 h in the robotic platform at 30 °C 1000 rpm. OD_{600} was measured online every hour for each well in each strain ($n = 8$). Numbers refer to "*prsA*.promoter". Therefore a) MTP3, b) MTP4, c) MTP5, d) MTP5 IPTG. Number resolution for PrsA-molecules is listed in Table S2.

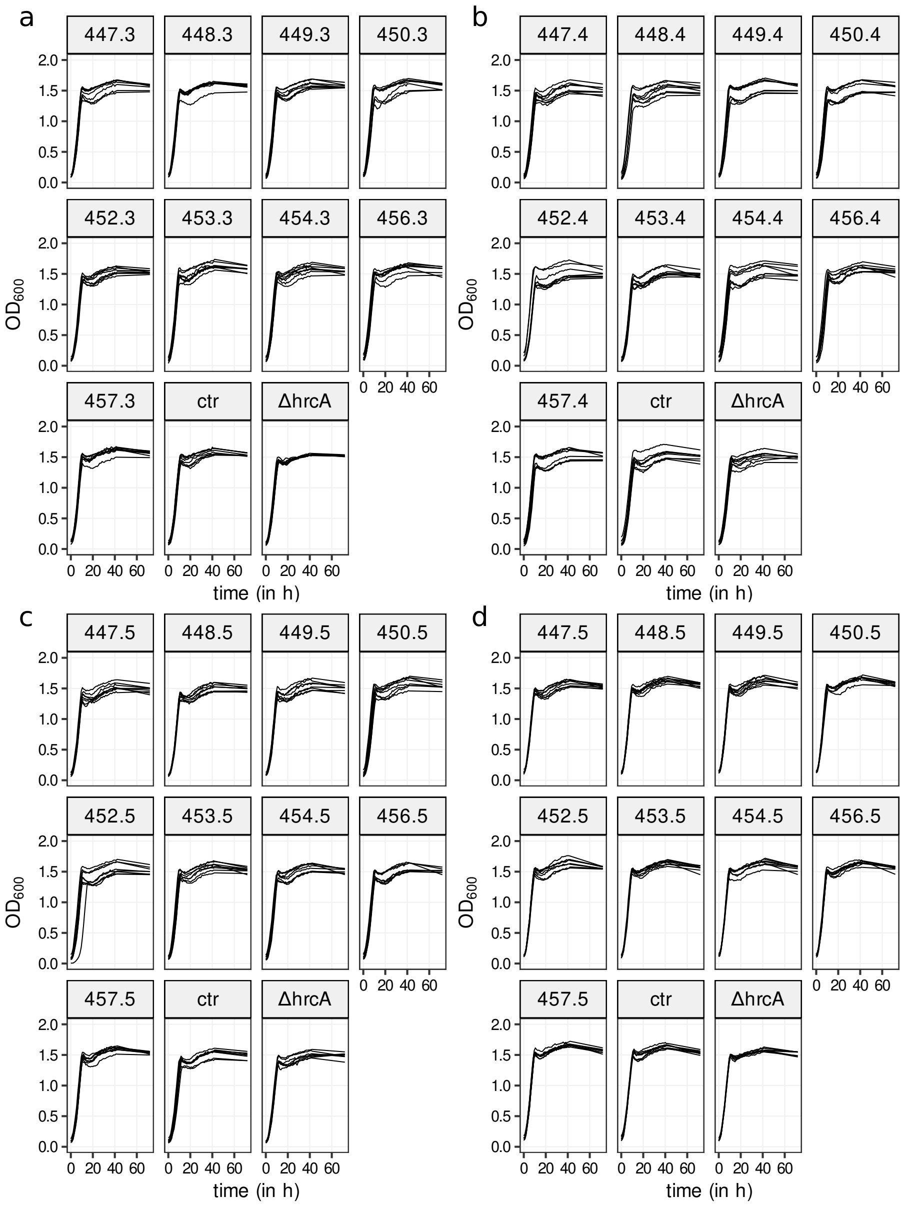

Figure S 9: All growth curve data gathered during cultivation of PrsA test in *amyS* strains. S19006 *amyS* was transformed with *prsA*-cassettes. For each *prsA*, eight different transformed colonys were picked and cultivated for 72 h in the robotic platform at 30 °C 1000 rpm. OD_{600} was measured online every hour for each well in each strain ($n = 8$). Numbers refer to "*prsA*.promoter". Therefore a) MTP3, b) MTP4, c) MTP5, d) MTP5 IPTG. Number resolution for PrsA-molecules is listed in Table S2.

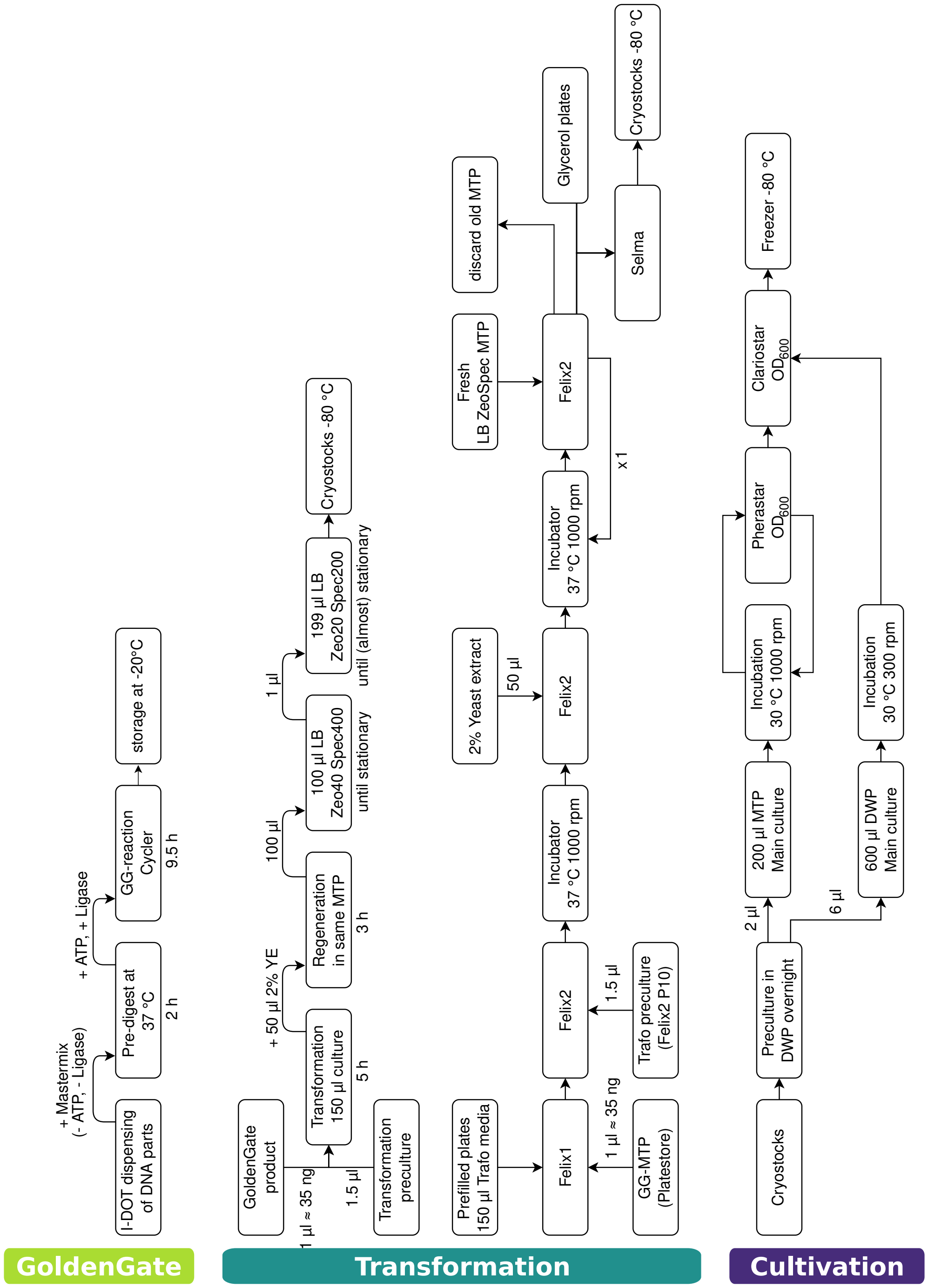

Figure S 10: Chart of the workflow for automated cloning, transformation- and cultivation. The workflow is split into three parts, after any of which a pausing of the process is possible. Felix1 & Felix2 are the pipette based liquid handling robots, while I-DOT is a contactless nanoliter dispenser and Selma is a semi-manual operated 96-well pipettor which is not integrated into the robotic platform. Pherastar is the MTP reader integrated into the platform, while Clariostar is the manually operated plate reader.

3 Supplemental Tables

Table S 1: Genotypes of used *Bacillus subtilis* strains relevant to this study.

Strain name	Parent strain	Relevant genotype
S19001	6051HGW	Δ*nprE*, Δ*aprE*; Δ*epr*, Δ*mpr*, Δ*nprB*, Δ*vpr*, Δ*bpr*, Δ*sacA*::(*ZeoR*, P_{xylA}-*cre*, *xylR*, P_{spac}-*comS*, *lacI*), Δ*sigE*/*spoIIGA*::*lox72*
S19002	6051HGW	Δ(*tasA-yqxM*)::*lox72*, Δ(*ldh-lctP*)::*lox72*, Δ(*endB-ndoA*)::*lox72*, Δ(*yjpA-spoIISA*)::*lox72*, Δ(*epsO-epxA*)::*lox72*, Δ(*srfAA-srfAD708*)::*lox72*, Δ(*ydzT24-ydjC*)::*lox72*, Δ(*pksA-pksR7611*)::*lox72*, Δ(*ppsE-ppsA*)::*lox72*, Δ(*yodU-ypqP540*)::*lox72*, Δ*lytC*::*lox72*, Δ*sacA*::(*ZeoR*, $P_{xylA-cre}$, *xylR*, $P_{spac-comS}$, *lacI*)
S19003	6051HGW	Δ*spoIIGA*::*lox72*, Δ*lytC*::*lox72*, Δ*srfAA-AD*::*comS-lox72*, Δ(*endB-ndoA*)::*lox72*
S19004	-	Δ*nprE*, Δ*aprE*, Δ*sigF*::*lox72*
S19005	-	Δ*nprE*, Δ*aprE*, Δ*epr*,Δ*mpr*, Δ*nprB*, Δ*vpr*, Δ*bpr*, Δ*sigF*
S19006	-	Δ*nprE*, Δ*aprE*,Δ*epr*, Δ*mpr*, Δ*nprB*, Δ*vpr*, Δ*bpr*, Δ*sigF*, Δ*xpf*, Δ*lytC*, Δ*sdpC*, Δ*skfA*
S19034	S19002	Δ*aprE*::*lox72*, Δ*nprE*::*lox72*, Δ*mpr*::*lox72*, Δ*nprB*::*lox72*, Δ*vpr*::*lox72*, Δ*bpr*::*lox72*, Δ*epr*::*lox72*
S19038	S19034	Δ*estA*::$P_{BASF\#3}$-*prsA#447-lox72*

Table S 4: Oligonucleotides used in this work.

Name	Description	Sequence 5' - 3'
o10032_amyE-Seq1_fw	*amyE* sequencing	CGTAAAAGTGCGGGAGG
o10472_hrcA_front_fw	*hrcA* locus sequencing	TAGTGATGAGCAAGATGGAAGC
o10475_hrcA_back_rv	*hrcA* locus sequencing	TTGATTCACTTTGACCATGGAAG
o10482_estA_fw	*prsA* cassette amplification	GCCTTCTAACCTGTACAATGC
o10483_estA_rv	*prsA* cassette amplification	CTTCTTTTGCCGCTAGTTCC
o11308_SpecRseq	*specR* sequencing	CCATTAGAACATAGGGAGAGAATTTTG
o11309_pDR111seq	*specR* sequencing	CAAGGCGTGTCTCACCAG
o11379_SpecR_Seq	*specR* sequencing	GTGGGAAGGACTATATTCAAAGGTG
o11380_SpecR_Seq	*specR* sequencing	GCTGTTAATGCGTAAACCACC

Continued on next page

Table S 4 – continued from previous page

Name	Description	Sequence 5' - 3'
o14013_pJET1.2-Seq-fwd	pJET1.2 insert sequencing	CGACTCACTATAGGGAGAGCGGC
o14014_pJET1.2-Seq-rev	pJET1.2 insert sequencing	AAGAACATCGATTTTCCATGGCAG
o14068_pJK179-Seqlox66-fw	knockout plasmid assembly check	CACTGGCCGTCGTTTTAC
o14089_Spec-mid-fw	SSS amplification	CAATAGCCAAATCAGGATCATAGC
o14090_Spec-mid-rv	SSS amplification	GGACAAATTCAGGAACCAAGC
o14127_Seq-lox71	knockout plasmid assembly check	CACTGCCCGCTTTCCAG
o14249_SSS-l71_fw	SSS amplification	ATCACGAATTGGATCCTCGAG
o14250_SSS-l66_rv	SSS amplification	ATCAGCGCTTCACCAATTCATC
o19001_vpr_rv	knockout plasmid cassette check	GCGCTGGCTATATGTTTGCC
o19002_aprE_rv	knockout plasmid cassette check	TCCTGATAACGCGAGACAGC
o19003_aprE_fw	knockout plasmid cassette check	CCTTGCAAATCGGATGCCTG
o19004_vpr_fw	knockout plasmid cassette check	CTGCCAAAAAGAAGCGGAGG
o19005_aprE_back_oh_fw	deletion of *aprE*	AGATCTTCCGGATGGCTCGAGTTTTTCAGCAAGATGCCATTATGTCATGAAGCAC
o19006_aprE_back_oh_rv	deletion of *aprE*	TACGAACGGTAGGCCTCGAGGATCCAATTCGTGATTAATCAACGTACAAGCAGCTG
o19007_aprE_fr_oh_fw	deletion of *aprE*	CGGTAGGCCTCTAGATGAATTGGTGAAGCGCTGATCTCTCGCTATTTCCGTAGAG
o19008_aprE_fr_oh_rv	deletion of *aprE*	AGCTGAGAATATTGTAGGAGATCTTCTAGAAAGATGACACAGAAGAAAACGTTGG
o19009_aprE_seq_fw	seqencing of *aprE* locus	TATGTTACAGGAATTGGGGC
o19010_aprE_seq_rv	seqencing of *aprE* locus	GTGTTAAACATTTTGCCCCG
o19011_nprE_back_oh_fw	deletion of *nprE*	AGATCTTCCGGATGGCTCGAGTTTTTCAGCAAGATGAGACAGCTGGATTTGTGC
o19012_nprE_back_oh_rv	deletion of *nprE*	TACGAACGGTAGGCCTCGAGGATCCAATTCGTGATTCTCTCTCAGCTGAAACGAC

Continued on next page

Table S 4 – continued from previous page

**Name**	**Description**	**Sequence 5' - 3'**
o19013_nprE_fr_oh_fw	deletion of *nprE*	CGGTAGGCCTCTAGATGAATTGGTGAAGCGCTGATATCCCCCTTTTTGAAAATACTGA
o19014_nprE_fr_oh_rv	deletion of *nprE*	AGCTGAGAATATTGTAGGAGATCTTCTAGAAAGATGTAAGCGCTGGTGAAGTTTG
o19015_nprE_seq_fw	seqencing of *nprE* locus	CCGCTCCAAGAATGACATAC
o19016_nprE_seq_rv	seqencing of *nprE* locus	CAGCATATAGTGAAAAGCCGT
o19017_bpr_front_oh_fw	deletion of *bpr*	AGATCTTCCGGATGGCTCGAGTTTTTCAGCAAGATAAAAGCAATTTCCAGCCCG
o19018_bpr_front_oh_rv	deletion of *bpr*	TACGAACGGTAGGCCTCGAGGATCCAATTCGTGATAGCGAAGGAAGCAATAGGG
o19019_bpr_back_oh_fw	deletion of *bpr*	CGGTAGGCCTCTAGATGAATTGGTGAAGCGCTGATACTTCCTCGACAAATTAAGCAG
o19020_bpr_back_oh_rv	deletion of *bpr*	AGCTGAGAATATTGTAGGAGATCTTCTAGAAAGATATAAACAACCAGCTGATCGG
o19021_bpr_seq_fw	seqencing of *bpr* locus	AAGGATCTGCTTTGATGGGT
o19022_bpr_seq_rv	seqencing of *bpr* locus	GCTGACTCGTTCCTCATATTG
o19023_epr_front_oh_fw	deletion of *epr*	AGATCTTCCGGATGGCTCGAGTTTTTCAGCAAGATGTGTAAACGGCACCTCAAAC
o19024_epr_front_oh_rv	deletion of *epr*	TACGAACGGTAGGCCTCGAGGATCCAATTCGTGATTCATGGGCAGGGTTGTTTAG
o19025_epr_back_oh_fw	deletion of *epr*	CGGTAGGCCTCTAGATGAATTGGTGAAGCGCTGATACCACACACAACCTTTTTCC
o19026_epr_back_oh_rv	deletion of *epr*	AGCTGAGAATATTGTAGGAGATCTTCTAGAAAGATGCAGGTTCTCAATGGCATG
o19027_epr_seq_fw	seqencing of *epr* locus	GCGACAAATAAGCCCTTG
o19028_epr_seq_rv	seqencing of *epr* locus	TGCTCCGAAGATAATCTGG

Continued on next page

Table S 4 – continued from previous page

Name	Description	Sequence 5' - 3'
o19029_nprB_front_oh_fw	deletion of *nprB*	AGATCTTCCGGATGGCTCGAGTTTTTCAGCAAGATGTTCCTCAATGCGCTTGTC
o19030_nprB_front_oh_rv	deletion of *nprB*	TACGAACGGTAGGCCTCGAGGATCCAATTCGTGATGCAAAACACCACATCCTTCC
o19031_nprB_back_oh_fw	deletion of *nprB*	CGGTAGGCCTCTAGATGAATTGGTGAAGCGCTGATCTGTCGGCATTCTATGAGC
o19032_nprB_back_oh_rv	deletion of *nprB*	AGCTGAGAATATTGTAGGAGATCTTCTAGAAAGATTGCTGCCGATCTCTGTATC
o19033_nprB_seq_fw	seqencing of *nprB* locus	TTGTCAAGATTTCGCGAGTC
o19034_nprB_seq_rv	seqencing of *nprB* locus	GCCAATGAAGTGAAGGAGGA
o19035_vpr_front_oh_fw	deletion of *vpr*	AGATCTTCCGGATGGCTCGAGTTTTTCAGCAAGATCATCCCTCCGCTTCTTTTTG
o19036_vpr_front_oh_rv	deletion of *vpr*	TACGAACGGTAGGCCTCGAGGATCCAATTCGTGATTCAATGTGTTTCCCCCTTTG
o19037_vpr_back_oh_fw	deletion of *vpr*	CGGTAGGCCTCTAGATGAATTGGTGAAGCGCTGATAAAGATCAGTCAGCAAACGC
o19038_vpr_back_oh_rv	deletion of *vpr*	AGCTGAGAATATTGTAGGAGATCTTCTAGAAAGATCACCGTTTTCCGAATCTGAC
o19039_vpr_seq_fw	seqencing of *vpr* locus	TACGCTGAGCCGAATAGAC
o19040_vpr_seq_rv	seqencing of *vpr* locus	CTGAATGACGGTGGTAAGC
o19041_wprA_front_oh_fw	deletion of *wprA*	AGATCTTCCGGATGGCTCGAGTTTTTCAGCAAGATGGGGAACATATATGACACACCT
o19042_wprA_front_oh_rv	deletion of *wprA*	TACGAACGGTAGGCCTCGAGGATCCAATTCGTGATTCATGTTATCCCTCCTGCAA
o19043_wprA_back_oh_fw	deletion of *wprA*	CGGTAGGCCTCTAGATGAATTGGTGAAGCGCTGATGCAAGGAAGCAAAAGTTGTTG

Continued on next page

Table S 4 – continued from previous page

Name	Description	Sequence 5' - 3'
o19044_wprA_back_oh_rv	deletion of *wprA*	AGCTGAGAATATTGTAGGAGATCTTCTAGAAAGATATCCTCATTGAAGACGGCAT
o19045_wprA_seq_fw	seqencing of *wprA* locus	ATAAAACTGGAGGGCGGAC
o19046_wprA_seq_rv	seqencing of *wprA* locus	GAGACAGCATGGATGAAACG
o19047_mpr_front_oh_fw	deletion of *mpr*	AGATCTTCCGGATGGCTCGAGTTTTTCAGCAAGATTGTTTGGTGTTGAGCTGTTC
o19048_mpr_front_oh_rv	deletion of *mpr*	TACGAACGGTAGGCCTCGAGGATCCAATTCGTGATTCATTTTGTCATCTCCCTCCT
o19049_mpr_back_oh_fw	deletion of *mpr*	CGGTAGGCCTCTAGATGAATTGGTGAAGCGCTGATGGGTGACGAACGATGTATTC
o19050_mpr_back_oh_rv	deletion of *mpr*	AGCTGAGAATATTGTAGGAGATCTTCTAGAAAGATAGGATAACGCCCCTTTTGT
o19051_mpr_seq_fw	seqencing of *mpr* locus	AGAGCAGCAAATAGAAGAAGC
o19052_mpr_seq_rv	seqencing of *mpr* locus	ATCATATGCCTGGTAAAAGCG
o19053_estA_seq_fw	sequencing of *estA* locus	TTCGATGAGACCTTCCACAG
o19054_estA_HomA_rv	sequencing of *prsA* cassettes	CAGATTTCTCCGTATTGGCG
o19055_loxSpecR_fw	sequencing of *prsA* cassettes	AGAGGCGGCCTTATTCAAAT
o19056_SpecR_fw	sequencing of *prsA* cassettes	AGCAGTTCGTAGTTATCTTGGA
o19057_estA_seq_rv	sequencing of *estA* locus	ACTGTGAAGGCATACTCCAC
o19058_prsA_seq_fw	sequencing of *prsA* cassettes	CGCCAATACGGAGAAATCTG
o19059_prsA_seq_rv	sequencing of *prsA* cassettes	ATTTGAATAAGGCCGCCTCT
o19060_estA_seq_fw	sequencing of *estA* locus	AGGACCAATAATGACCTCTGA
o19061_estA_seq_rv	sequencing of *estA* locus	ATGATATGGCTGCGAAAACC

Table S 2: Genetic material used in this work.

**Promoters**		
P_{syn2}	Constitutive promoter (high strength for enzyme expression)	
P_{veg8pb}	Constitutive promoter (weak-medium strength for enzyme expression)	
P_{secA}	Constitutive promoter	
P_{syn_weak}	Constitutive promoter (weak-medium strength for enzyme expression)	
P_{spac}	Inducible promoter	
**Amylases**	**Organism**	
amyB	*Bacillus licheniformis*	
amyS	*Geobacillus stearothermophilus*	
ALBA	*Bacillus sp.*	
SP722	*Bacillus sp.*	
BAN	*Bacillus amyloliquefaciens*	
707	*Bacillus sp.*	
Hybrid amylase	Hybrid from 707 and BAN	
TS-23	*Bacillus sp.*	
AA1-10	*Bacillus sp.*	
amyM	*Bacillus amyloliquefaciens*	
amy0354	proprietary metagenome data	
amy0355	proprietary metagenome data	
amy0356	proprietary metagenome data	
amy0365	proprietary metagenome data	
amy0379	proprietary metagenome data	
amy0381	proprietary metagenome data	
amy0386	proprietary metagenome data	
amy0392	proprietary metagenome data	
amy2405	proprietary metagenome data	
***prsA*#**	**Organism**	pI
447	*Bacillus licheniformis*	5.31
448	*Bacillus amyloliquefaciens XH7*	9.52
449	*Bacillus megaterium DSM319*	9.08
450	*Bacillus methanolicus MGA3*	9.05
452	*Bacillus pumilus SAFR032*	8.84
453	*Geobacillus stearothermophilus*	6.27
454	*Bacillus subtilis 168*	9.21
456	*Bacillus lentus*	4.49
457	*Bacillus halmapalus*	4.07
***Signal Peptides*#**	**Organism**	
AmyL	*B. licheniformis*	
YdjM	*B. subtilis*	
CwlS	*B. subtilis*	
YvcE	*B. subtilis*	
PhoD	*B. subtilis*	

Table S 3: Deletion plasmids used in this work.

**Deletion plasmids**	
p19002	*pJET1.2-aprE 5'homology-lox71-six site-specR-six site-lox66-aprE 3'homology*
p19003	*pJET1.2-nprE 5'homology-lox71-six site-specR-six site-lox66-nprE 3'homology*
p19004	*pJET1.2-bpr 5'homology-lox71-six site-specR-six site-lox66-bpr 3'homology*
p19005	*pJET1.2-epr 5'homology-lox71-six site-specR-six site-lox66-epr 3'homology*
p19006	*pJET1.2-nprB 5'homology-lox71-six site-specR-six site-lox66-nprB 3'homology*
p19007	*pJET1.2-vpr 5'homology-lox71-six site-specR-six site-lox66-vpr 3'homology*
p19008	*pJET1.2-wprA 5'homology-lox71-six site-specR-six site-lox66-wprA 3'homology*
p19009	*pJET1.2-mpr 5'homology-lox71-six site-specR-six site-lox66-mpr 3'homology*

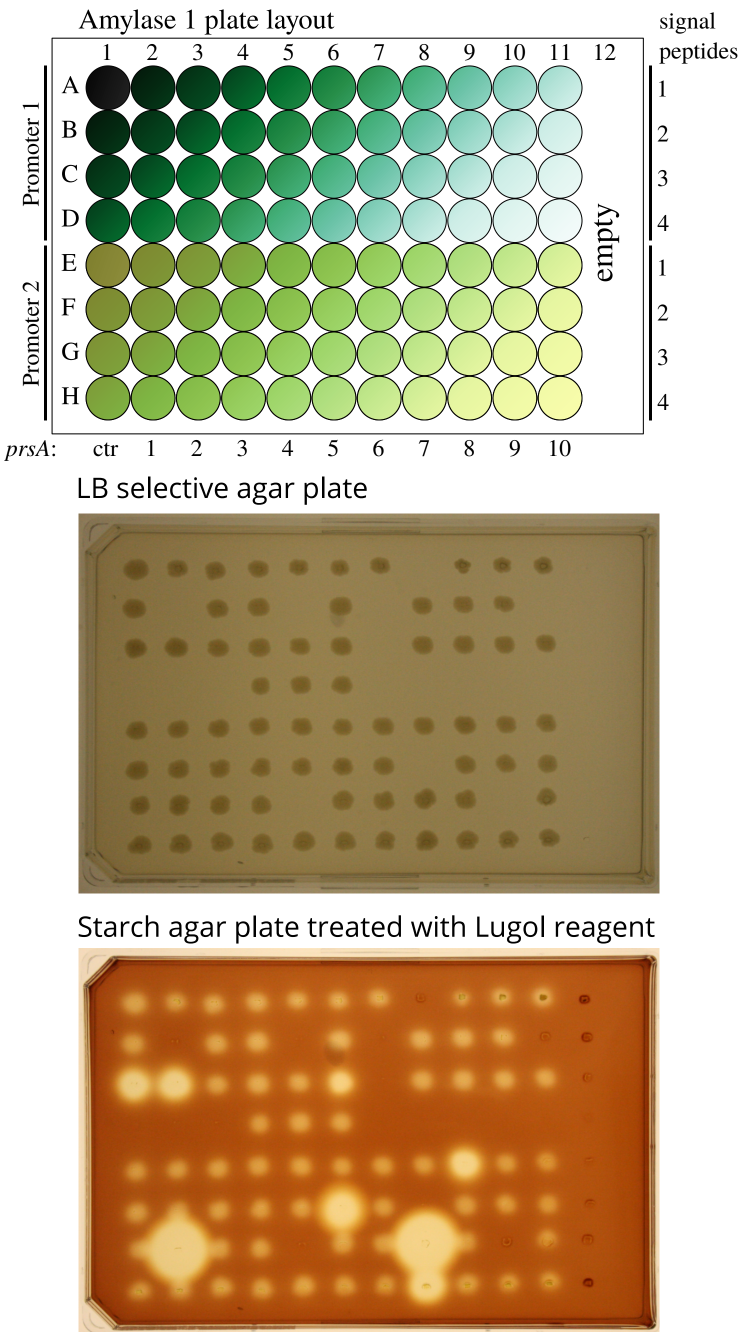

Figure S 11: Exemplary evaluation of first automated transformation using starch plates for a full factorial of a combination of an amylase with two different promoters and 11 signal peptides transformed into 11 different PrsA background strains. Small white spots on the starch plate are native AmyE-hydrolysis.

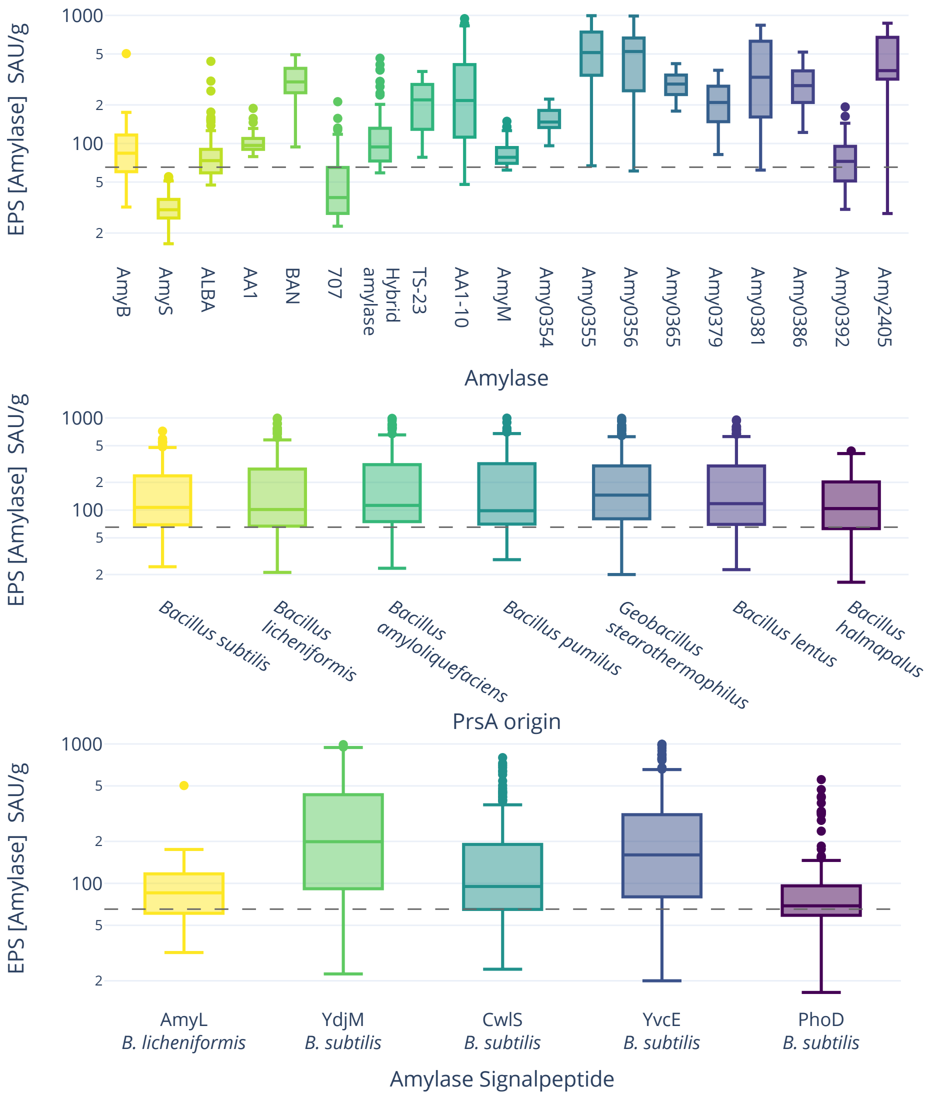

Figure S 12: Boxplots of end point determination of amylase activity (SAU/g: Standardized Amylase Units per gramm). A) amylase activity plotted per signal peptide resolved by PrsA chapperones present in the strains. B) Activity plotted per used amylase and resolved by PrsA molecule. The grey dashed lines are the background activities of the negative controls, which as all strains, contain the native amylase (AmyE) from *B. subtilils*.

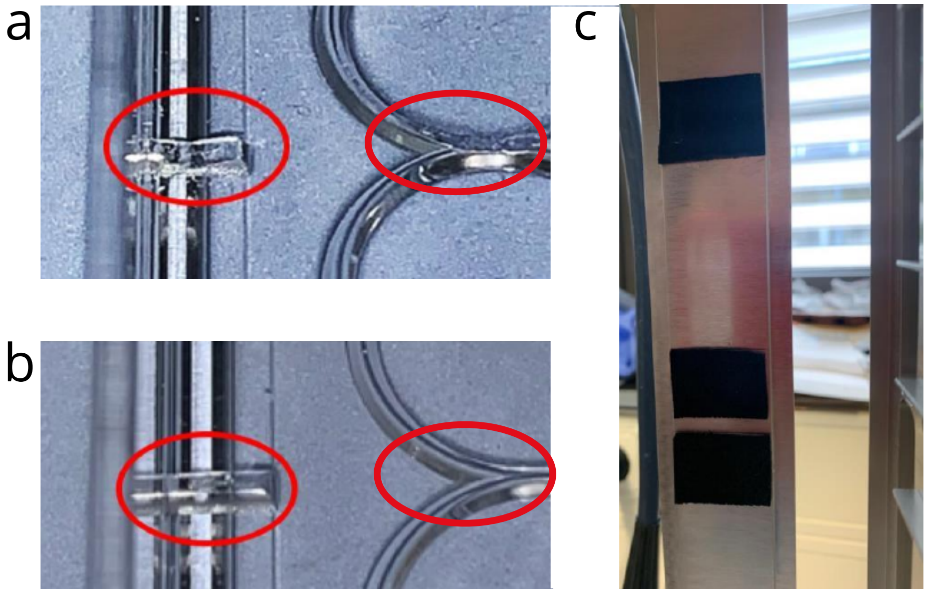

Figure S 13: Strong abrasion of plastic from the MTP lids occurred in the Cytomat2 shakers used (a) and could be massively reduced (b) by gluing cellular rubber with super glue to the back of the shakers of the Cytomat2. This solution was devised by engineering students from the TU Darmstadt and an extensive report is available upon reasonable request.

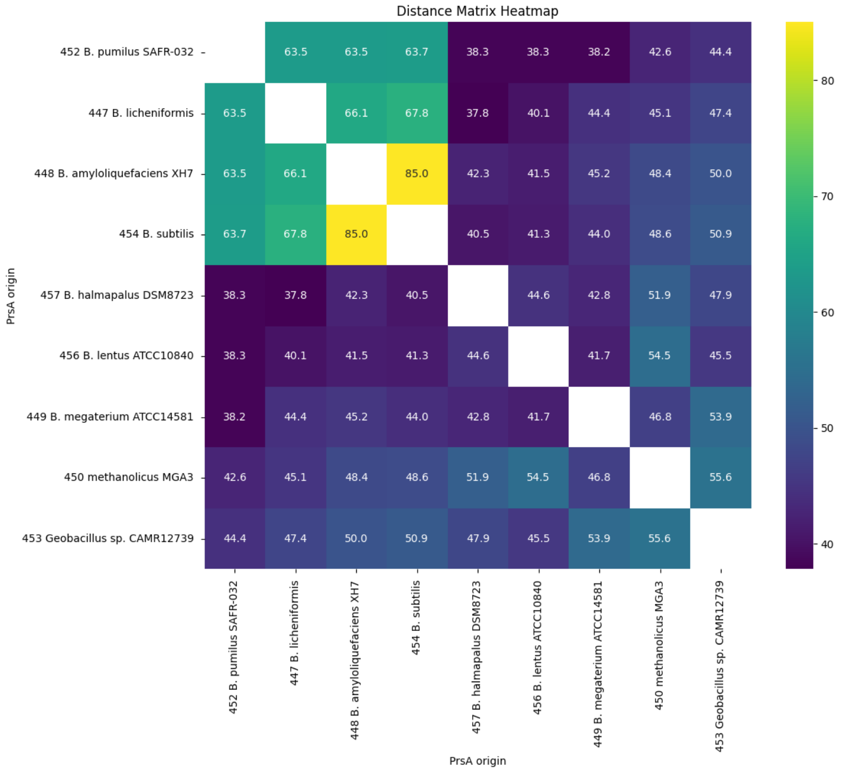

Figure S 14: Heatmap and distance matrix for the different PrsA molecules used. First the reference number used in the manuscript is listed followed by the originating species.

Table S 5: Analysis of non-sense frame-shifts leading to split proteins.

Gene	Synonym	Product
cgeD		protein involved in maturation of the outermost layer of the spore
comP		two-component sensor histidine kinase
csoR		repressor of copper utilization proteins
degA	iolQ	transcriptional repressor of iolX expression, transcriptional activator involved in the degradation of glutamine phosphoribosylpyrophosphate amidotransferase; control of scyllo-inositol utilization
dinB	bstG	bacillithiol S-transferase, nuclease inhibitor
dksA	yteA, YywB	RNA polymerase-binding transcription factor DksA, sporulation protein
dppE		dipeptide ABC transporter (dipeptide-binding lipoprotein)
glpG	yqgP, gluP	intramembrane protease
gtaB		UTP-glucose-1-phosphate uridylyltransferase
iscS	nifZ	cysteine desulfurase involved in tRNA thiolation
lplB		putative ABC transporter (permease)
lytF		gamma-D-glutamate-meso-diaminopimelate muropeptidase (major autolysin)
mltG		Endolytic murein transglycosylase, potential terminase for peptidoglycan polymerization
xylR		Xylose repressor
opuE		proline transporter
rsbRB		component of the piezosome (stressosome)
rsbRD		component of the piezosome (stressosome)
sigE		RNA polymerase sporulation-specific sigma-29 factor (sigma-E)
sipS		type I signal peptidase
sdpC		killing factor SdpC, non-sporulating cells
spoIIGA		protease processing pro-sigma-E
ybaK		sporulation protein
ybfG		putative pepdidoglycan binding protein
ydeL		putative PLP-dependent transcriptional regulator
yebC		putative transcriptional regulatory protein YebC
yflH		putative protein YflH, general stress protein
yhxA		similar to adenosylmethionine-8-amino-7-oxononanoate aminotransferase
yolD		hypothetical protein; phage SPbeta
yraG		forespore-specific sporulation protein, similar to spore coat protein
yrkF		putative rhodanese-related sulfur transferase
yrpD		putative lipoprotein
yulB	rhaR	transcriptional regulator (DeoR family) of the rhaEW-rhaR-rhaB-rhaM-rhaA operon
ywhK		factor interacting with DNA helicase PcrA
